# Irreducibility of limits of Galois representations of Saito–Kurokawa type

**DOI:** 10.1007/s40993-021-00265-x

**Published:** 2021-06-04

**Authors:** Tobias Berger, Krzysztof Klosin

**Affiliations:** 1 School of Mathematics and Statistics, University of Sheffield, Hicks Building, Hounsfield Road, Sheffield, S3 7RH UK; 2 Department of Mathematics, Queens College, City University of New York, 65-30 Kissena Blvd, Queens, NY 11367 USA

**Keywords:** Galois representations, The paramodular conjecture, *p*-adic Siegel modular forms, 11F80, 11F46

## Abstract

We prove (under certain assumptions) the irreducibility of the limit $\sigma _2$ of a sequence of irreducible essentially self-dual Galois representations $\sigma _k: G_{{\mathbf {Q}}} \rightarrow {{\,\mathrm{GL}\,}}_4(\overline{{\mathbf {Q}}}_p)$ (as *k* approaches 2 in a *p*-adic sense) which mod *p* reduce (after semi-simplifying) to $1 \oplus \rho \oplus \chi$ with $\rho$ irreducible, two-dimensional of determinant $\chi$, where $\chi$ is the mod *p* cyclotomic character. More precisely, we assume that $\sigma _k$ are crystalline (with a particular choice of weights) and Siegel-ordinary at *p*. Such representations arise in the study of *p*-adic families of Siegel modular forms and properties of their limits as $k\rightarrow 2$ appear to be important in the context of the Paramodular Conjecture. The result is deduced from the finiteness of two Selmer groups whose order is controlled by *p*-adic *L*-values of an elliptic modular form (giving rise to $\rho$) which we assume are non-zero.

## Introduction

In [8] the authors studied the modularity of abelian surfaces with rational torsion. Let *A* be an abelian surface over ${\mathbf {Q}}$, let *p* be a prime and suppose that *A* has a rational point of order *p*, and a polarization of degree prime to *p*. Then the (semi-simplified) action of $G_{{\mathbf {Q}}}:={{\,\mathrm{Gal}\,}}(\overline{{\mathbf {Q}}}/{\mathbf {Q}})$ on $A(\overline{{\mathbf {Q}}})[p]$ is of the form $1 \oplus \rho \oplus \chi$, for $\chi$ the mod *p* cyclotomic character. Assuming that $\rho$ is irreducible, Serre’s conjecture (Theorem of Khare-Wintenberger) implies that the mod *p* representation looks like the reduction of that of a Saito–Kurakawa lift of an elliptic modular form *f* of weight 2. If ${{\,\mathrm{End}\,}}(A)={\mathbf {Z}}$ then the *p*-adic Tate module of *A* gives rise to an irreducible *p*-adic Galois representation. The Paramodular Conjecture (formulated by Brumer and Kramer [15]) predicts that this representation should be isomorphic to the Galois representation attached to a weight 2 Siegel modular form of paramodular level which is not in the space of Saito–Kurokawa lifts. Establishing the modularity of *A* by a Siegel modular form therefore requires proving congruences between the Saito–Kurokawa lift *SK*(*f*) and “non-lifted type (G)” Siegel modular forms. The latter are cuspforms staying cuspidal under the transfer to ${{\,\mathrm{GL}\,}}_4$, and are expected to be exactly the forms whose associated *p*-adic representation is irreducible.

Such congruences for Saito–Kurokawa lifts have been proven by Brown, Agarwal and Li [1, 12, 14] for holomorphic Siegel modular forms of congruence level $\Gamma _0^{2}(N)$ and paramodular level $\Gamma _{\mathrm{para}}(N)$ for weights *k* larger than 6 (see [14] Corollary 6.15). With this new result [8] Theorem 10.2 can be generalized to allow ramification at a squarefree level *N*, and establishes a so-called $R=T$ result and the modularity of Fontaine–Laffaille representations that residually are of Saito–Kurokawa type (with an elliptic *f* of weight $2k-2$ for $k \ge 6$). Different type of congruences have also been constructed by Sorensen, see Sect. 5.2.

The methods used to prove these congruences unfortunately do not extend to weight $k=2$, the case of interest for the modularity of abelian surfaces. We propose to use *p*-adic families to prove the relevant congruences in weight 2 (albeit a priori only to a *p*-adic modular form—see below). For example, Skinner and Urban [32] proved that for an ordinary elliptic form *f* the $\Gamma _{\mathrm{para}}(N)$-level holomorphic Saito–Kurokawa lift *SK*(*f*) can be *p*-adically interpolated by a semi-ordinary (also called Siegel-ordinary) family. It is plausible that their arguments could be adapted for $\Gamma _0^2(N)$-level holomorphic Saito–Kurokawa lifts. Such *p*-adic families have also been studied by Kawamura [22] and Makiyama [24].

As part of a work in progress we construct (under some assumptions) another Siegel-ordinary *p*-adic family (of tame level either $\Gamma _0^{2}(N)$ or $\Gamma _{\mathrm{para}}(N)$) interpolating the type of congruences constructed by Brown or Sorensen. At classical weights $k \gg 0$ its points would correspond to irreducible *p*-adic Galois representations that are Siegel-ordinary (see Definition 2.3) and whose semi-simplified residual representation is the mod *p* representation associated to *SK*(*f*).

One could then use this family to approach weight 2 via weights $k \gg 0$, but $k \rightarrow 2$ *p*-adically. As points of weight 2 for such a family are critical (in the sense that the $U_p=U_{p,1} U_{p,2}$-slope is at least one and therefore does not satisfy the small slope condition in Theorem 7.1.1 of [2]; see Sect. 5.1 for definitions of $U_{p,1}$ and $U_{p,2}$) it is not clear whether this limit would correspond to a classical Siegel modular form.

In fact, modularity by *p*-adic Siegel modular forms was proved for certain abelian surfaces whose *p*-adic Galois representation is residually irreducible by Tilouine [38]. In a sense this paper provides a necessary ingredient to proving such *p*-adic modularity for the residually reducible case as explained below. Let us also mention that some strong potential modularity results in the residually irreducible situation have recently been proven in [11].

One potential problem is that while the *p*-adic Galois representations attached to the members of the family for $k \gg 0$ are irreducible this is not a priori clear of the limit. This property is on the one hand necessary for modularity purposes (as $T_pA\otimes {\mathbf {Q}}_p$ is irreducible). On the other hand it allows one then to feed these ingredients into a machinery similar to the one developed in [8] (modified appropriately for representations that are Siegel-ordinary instead of Fontaine-Laffaille) and under suitable conditions show that $T_pA$ and the limit Galois representation are in fact isomorphic, thus proving *p*-adic modularity of *A*.

In this paper we introduce a new way of proving that under certain assumptions the limit of irreducible Galois representations is itself irreducible. This method is based on finiteness of Selmer groups and while we only apply it here in our specific situation (i.e., when the representations are residually of Saito–Kurokawa type, as desired for proving the modularity of abelian surfaces with rational *p*-torsion) it is not difficult to see how it can be modified to work in other contexts, cf. our upcoming paper about a residually reducible $R=T$ result for ${{\,\mathrm{GL}\,}}_2$ in weight 1.

In other words, while our overarching goal is to provide ingredients to prove modularity of abelian surfaces as explained above, the theorems proven in this paper could in principle be treated completely independently as a result on limits of Galois representations. In particular, Siegel modular forms will be notably absent from our statements and their presence will manifest itself only through certain conditions imposed on the Galois representations. We thus consider a family (which is part of a “refined” rigid analytic family in the sense of Ballaïche–Chenevier—see Sect. 3) of irreducible 4-dimensional *p*-adic Galois representations $\sigma _k$ indexed by a set of integers $k >2$, $k\equiv 2$ (mod ($p-1$)) which approach 2 in the *p*-adic sense. Suppose that $tr \sigma _k$ converge *p*-adically to some pseudo-representation *T* when $k \rightarrow 2$. We require that for each *k* the representation $\sigma _k$ reduces to some mod *p* representation whose semi-simplification is isomorphic to $1 \oplus \chi \oplus \rho$ for an irreducible 2-dimensional representation $\rho$ and that it is crystalline and Siegel-ordinary. We are interested in conditions guaranteeing the irreducibility of *T*.

The basic idea is not difficult to explain. First we use the irreducibility of $\sigma _k$ to construct Galois stable lattices in their representation spaces so that infinitely many of the $\sigma _k$s reduce mod *p* to a non-semi-simple residual representation (whose semi-simplification is $1 \oplus \chi \oplus \rho$) with the same Jordan Holder factor as a subrepresentation and the same Jordan–Holder factor as a quotient. It is not possible to ensure that *all* $\sigma _k$ reduce to the same combination as $\overline{\sigma }_k$ has three Jordan–Holder factors. Indeed, in general Ribet’s Lemma only tells us that there are enough (non-split) extensions between different Jordan–Holder factors to guarantee connectivity of a certain graph—see Sect. 4—and absent any other assumptions (like for example lying in the Fontaine-Laffaille range which was used in Corollary 4.3 of [8]) there is no way to tell which extension will arise. However, as there are only finitely many such extensions possible, we get an infinite subsequence ${\mathcal {T}}$ of $\sigma _k$ with identical (non-split) reduction.

Now, if *T* was reducible, there are several ways in which it can split into the sum of irreducible pseudo-representations. Let us discuss here the case of three Jordan–Holder factors which can be regarded as the main result of this paper—see Theorem 3.3. In that case as $k\in {\mathcal {T}}$ approaches 2 (*p*-adically) the representations $\sigma _k$ become reducible modulo $p^{n_k}$ with $n_k$ tending to $\infty$. As the reduction of $\sigma _k$ is non-split, we conclude that $\sigma _k$ give rise to elements in a certain Selmer group of arbitrary high order. Using symmetries built into the Galois representation one shows that this Selmer group can only be one of two possibilities. Then the Main Conjecture of Iwasawa Theory gives us that the orders of these Selmer groups are controlled by specializations to weight 2 (at two different points) of a certain *p*-adic *L*-function. Hence to guarantee that these Selmer groups are finite (i.e., that *T* cannot be reducible) we impose a non-vanishing condition on these *L*-values. As we a priori do not know for which of the possible extensions we get the infinite subsequence ${\mathcal {T}}$ we need to control both of the *L*-values as above. See Sect. 4 for details.

Let us now state the main result of the paper. For an ordinary newform $g=\sum _{n=1}^{\infty } a_n(g)q^n$ of weight 2 let *L*(*g*, *s*) denote the standard *L*-function of *g* and let $L_p(g,2)$ be the *p*-adic *L*-value denoted by $L_p^{\mathrm{an}}(g, \omega ^{-1}, T=p)$ in Sect. 2 of [8]. Write *N* for the prime-to-*p* conductor of $\rho$.

### Theorem 1.1

Assume $N \ne 1$ and that $\rho |_{G_K}$ is absolutely irreducible for $K={\mathbf {Q}}(\sqrt{(-1)^{(p-1)/2}p})$. Suppose that $L(g,1)L_p(g,2)\ne 0$ for all *p*-ordinary newforms *g* of weight 2 and level dividing *Np* such that $a_{\ell }(g) \equiv tr \rho ({{\,\mathrm{Frob}\,}}_{\ell })$ mod $\varpi$ for all primes $\ell \not \mid Np$. Then *T* is not of Saito–Kurokawa type (i.e., it does not split into 3 Jordan–Holder factors).

A priori if *T* is reducible it could also split into 2 or 4 components and we deal with them in Sects. 3 and 6. We are able to rule out all of them, albeit for the reduction type dealt with in Sect. 6, the so called Yoshida type, our theorems require quite strong assumptions.

We would like to thank Adel Betina, Pol van Hoften, Chris Skinner, and Ariel Weiss for helpful discussions related to the topics of this article and Andrew Sutherland for the example in Sect. 5.2. We would also like to express our gratitude to the anonymous referee for their careful reading of the original manuscript and numerous helpful suggestions.

## Setup

Let *p* be an odd prime. Let *E* be a finite extension of ${\mathbf {Q}}_p$ with integer ring ${\mathcal {O}}$, uniformizer $\varpi$ and residue field ${\mathbf {F}}$. We fix an embedding $\overline{{\mathbf {Q}}}_p \hookrightarrow {\mathbf {C}}$. Write $\epsilon$ for the *p*-adic cyclotomic character and $\chi$ for its mod $\varpi$ reduction. Let *N* be a square-free positive integer with $p\not \mid N$. Let $\Sigma$ be the set of primes of ${\mathbf {Q}}$ consisting of *p* and the primes dividing *N*. We denote by $G_{\Sigma }$ the Galois group of the maximal Galois extension of ${\mathbf {Q}}$ unramified outside of the set $\Sigma$.

Consider a Galois representation $\rho : G_{\Sigma } \rightarrow {{\,\mathrm{GL}\,}}_2({\mathbf {F}})$ of which we assume that it is odd and absolutely irreducible of determinant $\chi$. Furthermore we assume that $\rho$ is ordinary and *p*-distinguished, in the sense that

2.1 $$\begin{aligned} \rho |_{D_p} \cong \left[ \begin{array}{ll} \eta ^{-1}\chi &{} * \\ &{}\eta \end{array} \right] , \end{aligned}$$

where $\eta$ is a non-trivial unramified character and that $\rho |_{I_p}$ is non-split. We further assume that $\rho$ is ramified at all primes dividing *N* and that $\rho |_{I_{\ell }}$ has a fixed line for all $\ell \mid N$ (or equivalently that *N* is the prime-to-*p*-part of the conductor of $\rho$).

Let $\tau : G \rightarrow {{\,\mathrm{GL}\,}}_n({\mathcal {O}})$ be an *n*-dimensional representation of a group *G* or $\tau : {\mathcal {O}}[G] \rightarrow {\mathcal {O}}$ be an *n*-dimensional pseudo-representation of *G*. For a definition of a pseudo-representation, its dimension and basic properties we refer the reader to Sect. 1.2.1 of [5]. However, let us only mention here that an *n*-dimensional pseudo-representation $\tau$ is called *reducible* if $\tau = \tau _1 + \tau _2$ for some pseudo-representations $\tau _1, \tau _2$ (each necessarily of dimension smaller than *n*). A pseudo-representation that is not reducible is called *irreducible*. In particular, if $\tau : G\rightarrow {{\,\mathrm{GL}\,}}_n({\mathcal {O}})$ is a representation, then $T:=tr \tau$ is an *n*-dimensional pseudo-representation and *T* is reducible if and only if $\tau$ is. Furthermore if $\tau$ is an *n*-dimensional pseudo-representation and $\tau = \sum _{i=1}^{r} \tau _i$ with each $\tau _i$ an irreducible pseudo-representation, then this decomposition as a sum of irreducible pseudo-representations is unique (up to reordering of the summands).

Now let $G=G_{\Sigma }$. By composing a representation or pseudo-representation $\tau$ with the reduction map ${\mathcal {O}}\rightarrow {\mathbf {F}}$ we obtain the *reduction* of $\tau$ which we will denote by $\overline{\tau }$. If $\tau$ is an *n*-dimensional representation valued in ${{\,\mathrm{GL}\,}}_n(E)$, one can always find a $G_{\Sigma }$-stable ${\mathcal {O}}$-lattice $\Lambda$ such that when we choose a basis of $E^n$ to be a basis of $\Lambda$ we obtain a representation $\tau _{\Lambda }$ valued in ${{\,\mathrm{GL}\,}}_n({\mathcal {O}})$. The isomorphism class of $\tau _{\Lambda }$ and also of its reduction $\overline{\tau }_{\Lambda }$ depends in general on the choice of $\Lambda$. However, the semi-simplification $\overline{\tau }^{\mathrm{ss}}_{\Lambda }$ (and hence also the pseudo-representation $tr \overline{\tau }_{\Lambda }$) is independent of $\Lambda$ and so it makes sense to drop $\Lambda$ from the notation.

### Lemma 2.1

Let $\tau : G_{\Sigma } \rightarrow {{\,\mathrm{GL}\,}}_n(E)$ be a continuous representation and let *V* be the representation space of $\tau$. Suppose that there exists a subspace $L \subset V$ of dimension $r \le n$ with the following two properties: *L* is stable under $G_{\Sigma }$ and $G_{\Sigma }$ acts on *L* via an irreducible representation $\psi : G_{\Sigma } \rightarrow {{\,\mathrm{GL}\,}}_r(E)$ with values in ${{\,\mathrm{GL}\,}}_r({\mathcal {O}})$. Let $\Lambda$ be a $G_{\Sigma }$-stable ${\mathcal {O}}$-lattice in *V* ($\Lambda \otimes _{{\mathcal {O}}}E=V$). Then $\Lambda$ has a rank *r* free ${\mathcal {O}}$-submodule which is stable under $G_{\Sigma }$ and on which $G_{\Sigma }$ acts via the representation $\psi$.

### Proof

Let $\Lambda '$ be a $G_{\Sigma }$ stable lattice in *L*. Then for some positive integer *s* we have that $\Lambda _0:=\varpi ^s\Lambda ' \subset \Lambda$. Then $\Lambda _0$ is clearly a rank *r* free ${\mathcal {O}}$-submodule of $\Lambda$ on which $G_{\Sigma }$ acts via $\psi$. $\square$

### Lemma 2.2

Let $\tau : G_{\Sigma } \rightarrow {{\,\mathrm{GL}\,}}_n(E)$ be an irreducible representation. Suppose that with respect to some $G_{\Sigma }$-stable ${\mathcal {O}}$-lattice $\Lambda$ of the representation space *V* of $\tau$ one has $$\overline{\tau }_{\Lambda } \cong \left[ \begin{matrix} \tau _1 &{} * \\ {} &{} \tau _2\end{matrix} \right] $$ for $\tau _i: G_{\Sigma }\rightarrow {{\,\mathrm{GL}\,}}_{r_i}({\mathbf {F}})$, $r_1+r_2=n$. Then there exists a $G_{\Sigma }$-stable ${\mathcal {O}}$-lattice $\Lambda '$ of the representation space *V* such that with respect to $\Lambda '$ we have $$\overline{\tau }_{\Lambda '} \cong \left[ \begin{matrix}\tau _1 \\ * &{}\tau _2\end{matrix} \right] $$.

### Proof

For $g \in G_{\Sigma }$ write $$\tau _{\Lambda }(g)=\left[ \begin{matrix} a_g &{} b_g \\ c_g &{} d_g \end{matrix} \right] $$. Then $c_g$ is an $r_2 \times r_1$ matrix whose entries we denote by $c_{ij}(g)$. Let $S=\{ g \in G_{\Sigma } \mid c_g \ne 0\}$. Irreducibility of $\tau$ guarantees that *S* is non-empty. For $g \in S$ set $m_g:= \min \{{{\,\mathrm{val}\,}}_{\varpi }(c_{ij}(g))\mid i,j such that c_{ij}(g) \ne 0\}$. Furthermore set $m = \min _{g \in S}m_g$ and note that $m\ge 1$ as $\overline{\tau }_{\Lambda }$ is upper-triangular. Then

$$\begin{aligned} \left[ \begin{matrix} 1 \\ &{} \varpi ^{-m}\end{matrix} \right] \left[ \begin{matrix} a_g &{} b_g \\ c_g &{} d_g \end{matrix} \right] \left[ \begin{matrix} 1 \\ &{} \varpi ^{m}\end{matrix} \right] = \left[ \begin{matrix} a_g &{} \varpi ^m b_g \\ \varpi ^{-m} c_g &{} d_g \end{matrix} \right] . \end{aligned}$$

$$\square $$

In this article we will be especially interested in 2-dimensional and 4-dimensional Galois representations that are *ordinary* in a sense that we now define.

### Definition 2.3

1. A Galois representation $\tau : G_{\Sigma } \rightarrow {{\,\mathrm{GL}\,}}_2(E)$ will be called *ordinary* if $$\tau |_{D_p} \cong \left[ \begin{matrix} \psi ^{-1} \epsilon ^{k-1}&{}* \\ &{} \psi \end{matrix} \right] $$ for some positive integer *k* and some unramified character $\psi$.
2. A Galois representation $\tau : G_{\Sigma } \rightarrow {{\,\mathrm{GL}\,}}_4(E)$ will be called *Siegel-ordinary* if
  $$\begin{aligned} \tau |_{D_p} \cong \left[ \begin{matrix} \psi ^{-1}\epsilon ^{2k-3} &{} * &{} * &{} * \\ &{} * &{} * &{} * \\ &{}*&{}*&{}*\\ &{}&{}&{}\psi \end{matrix} \right] , \end{aligned}$$
  for some positive integer *k* and some unramified Galois character $\psi$.
3. A Galois representation $\tau : G_{\Sigma } \rightarrow {{\,\mathrm{GL}\,}}_4(E)$ will be called *Borel-ordinary* if
  $$\begin{aligned} \tau |_{D_p} \cong \left[ \begin{matrix} \psi ^{-1}\epsilon ^{2k-3} &{} *&{} * &{} * \\ &{} \phi ^{-1} \epsilon ^{k-1} &{} * &{} * \\ &{} &{}\phi \epsilon ^{k-2}&{}*\\ &{}&{}&{}\psi \end{matrix} \right] , \end{aligned}$$
  for some positive integer *k* and some unramified Galois characters $\psi$ and $\phi$.

For later it will be useful to introduce the following notation. If $\alpha \in E^{\times }$, then the unramified character from $D_p$ to $E^{\times }$ that takes the arithmetic Frobenius to $\alpha$ will be denoted by $\phi _{\alpha }$.

## Irreducibility

### Main assumptions

Assume we have a *p*-adic family of Galois representations in the sense of [5], i.e. we have a rigid analytic space *X* over ${\mathbf {Q}}_p$ and a 4-dimensional pseudo-representation ${\mathbf {T}}: G_{\Sigma } \rightarrow {\mathcal {O}}(X)$. We denote by $\sigma _x:G_{\Sigma } \rightarrow {{\,\mathrm{GL}\,}}_4(E(x))$ (for some finite extension *E*(*x*) of ${\mathbf {Q}}_p$) the semi-simple representation of $G_{\Sigma }$ whose trace is the evaluation ${\mathbf {T}}_x$ of ${\mathbf {T}}$ at $x \in X$ (for existence see [35], Theorem 1). We are interested in the case when the family satisfies nice *p*-adic Hodge properties for all points in a Zariski dense set $Z \subset X$ and want to deduce properties at a point $x_0 \in X \backslash Z$, in particular to control the ramification at *p* of the corresponding Galois representation. The reader should think of *X* as (an affinoid subdomain of) an eigenvariety parametrizing Siegel modular forms. We therefore also assume the existence of a weight morphism $w: X \rightarrow {\mathcal {W}}$, where ${\mathcal {W}}$ is the rigid analytic space over ${\mathbf {Q}}_p$ such that ${\mathcal {W}}({\mathbf {C}}_p)={{\,\mathrm{Hom}\,}}_{\mathrm{cts}}(({\mathbf {Z}}_p^{\times })^2, {\mathbf {C}}_p^{\times })$.

More precisely, assume that we have data $(X, {\mathbf {T}}, \{\kappa _n\}, \{F_n\}, Z)$, a refined family in the sense of [5] Definition 4.2.3, where $n=1, \ldots 4$ and $\kappa _n$ and $F_n$ are analytic functions in ${\mathcal {O}}(X)$. For $z \in Z$ we have $0=\kappa _1(z)< \kappa _2(z)<\kappa _3(z) < \kappa _4(z)$ are the Hodge–Tate weights of $\sigma _z$. Different to [5] we use arithmetic Frobenius conventions throughout, in particular we say that ${\mathbf {Q}}_p(1)$ has weight 1 and Sen polynomial $X-1$. For the unramified character $\phi _\alpha$ defined above the eigenvalue of crystalline Frobenius on $D_{\mathrm{cris}}(\phi _{\alpha })$ equals $\alpha$.

The case of interest to us is where for a point *z* of weight $w(z)=(w_1, w_2)$ with $w_1\ge w_2$ we have $\kappa _2(z) = w_2-2$, $\kappa _3(z)=w_1-1$ and $\kappa _4(z) = w_1+w_2-3$. We assume $\sigma _z$ is crystalline and the eigenvalues of $\varphi$ on $D_{\mathrm{cris}}(\sigma _z)$ are given by $(p^{\kappa _1(z)}F_1(z), \ldots , p^{\kappa _4(z)}F_4(z))$. Furthermore, suppose there exists an involution $\tau :{\mathcal {O}}(X)[G_{\Sigma }] \rightarrow {\mathcal {O}}(X)[G_{\Sigma }]$ given by $\tau (g) = \Phi (g)g^{-1}$ for some character $\Phi : G_{\Sigma } \rightarrow {\mathcal {O}}(X)^{\times }$ with $\Phi |_{D_p} = \epsilon ^{\kappa _4(z)}$ such that ${\mathbf {T}}\circ \tau ={\mathbf {T}}$.

We also assume that for $z \in Z$ the representation $\sigma _z|_{D_p}$ is Siegel-ordinary, i.e. that

$$\begin{aligned} \sigma _z|_{D_p} \cong \left[ \begin{matrix} \psi ^{-1}\epsilon ^{\kappa _4(z)} &{} * &{} * &{} * \\ &{} * &{} * &{} * \\ &{}*&{}*&{}*\\ &{}&{}&{}\psi \end{matrix} \right] . \end{aligned}$$

This is equivalent to demanding that $|F_1(z)|=1$ and then $\psi =\phi _{F_1(z)}$. The existence of $\tau$ then implies that $F_4(z)=F_1(z)^{-1}$. In addition we assume that $\sigma _z$ is *p*-distinguished, i.e., $\overline{\psi } \ne 1$.

Fix $x_0\in X\setminus Z$ of weight $w(x_0)=(2,2)$ and from now we reserve the notation *E* for the field $E(x_0)$ and denote by ${\mathcal {O}}$ the ring of integers in *E* with uniformizer $\varpi$ and residue field ${\mathbf {F}}$. Put $T={\mathbf {T}}_{x_0}$ and $\sigma _2:= \sigma _{x_0}$. We assume that $T \equiv 1 + tr (\rho ) + \chi \mod \varpi$ for $\rho$ as in Sect. 2 and that $F_2(x_0) \ne 0$.

Let ${\mathcal {S}}$ be a sequence of integers $k \equiv 2$ (mod $p^{m_k-1}(p-1)$) with $m_k \rightarrow \infty$ as $k \rightarrow \infty$. We assume there exists a sequence of points $z_k \in Z$ converging to $x_0$ with $w(z_k)=(k,k)$ for $k \in {\mathcal {S}}$. Denote the corresponding family of Galois representations $\sigma _k:=\sigma _{z_k}: G_{\Sigma } \rightarrow {{\,\mathrm{GL}\,}}_4(E_k)$, where we set $E_k:= E(z_k)$. Extending $E_k$ if necessary we may assume that ${\mathcal {O}}\subset {\mathcal {O}}_k$, where ${\mathcal {O}}_k$ is the ring of integers of $E_k$ with uniformizer $\varpi _k$. Then we define $n_k \in {\mathbf {Z}}_{\ge 0}$ to be the largest integer *n* such that $tr \sigma _k \equiv T$ mod $\varpi ^{n}$. Note the convergence $z_k \rightarrow x_0$ implies $n_k \rightarrow \infty$ as $k\rightarrow \infty$ but approaches 2 *p*-adically.

We assume that for each $k\in {\mathcal {S}}$ the representations $\sigma _k$ have the following properties (of which (2), (3) and (5) follow from the assumption made on ${\mathbf {T}}$ and so does (4) for $k\gg 0$, but we record them here again for the ease of reference):

1. $$\sigma _k$$ is irreducible,
2. $$\det \sigma _k = \epsilon ^{4k-6}$$,
3. $$\sigma _k^{\vee } \cong \sigma _k(3-2k)$$,
4. $$\overline{\sigma }_k^{\mathrm{ss}}\cong 1 \oplus \rho \oplus \chi $$,
5. $\sigma _k|_{D_p}$ is crystalline with weights $2k-3, k-1, k-2, 0$ and $\sigma _k$ is Siegel-ordinary at *p*, i.e.,
  $$\begin{aligned} \sigma _k|_{D_p} \cong \left[ \begin{matrix} \phi _{\beta _k}^{-1}\epsilon ^{2k-3} &{} * &{} * &{} * \\ &{} * &{} * &{} * \\ &{}*&{}*&{}*\\ &{}&{}&{}\phi _{\beta _k}\end{matrix} \right] , \end{aligned}$$
  for $\beta _k \in {\mathcal {O}}_k^{\times }$ and we assume that $\beta _k \not \equiv 1$ mod $\varpi _k$, i.e., $\overline{\sigma }_k$ is *p*-distinguished;
6. If $\ell \in \Sigma - \{p\}$ then $\sigma _k|_{I_{\ell }}$ is unipotent (see Remark 4.5 for a potential weakening of this condition).

We refer the reader to Theorem 5.1 for a relation between these properties of $\sigma _k$ and Siegel modular forms.

#### Lemma 3.1

We have

(i) $T|_{D_p}=\phi _{\beta }^{-1} \epsilon + \phi _{\beta } +tr \gamma$ for $\beta = F_1(x_0)$ and a continuous representation $\gamma :D_p \rightarrow \mathrm{GL}_2({\mathcal {O}})$.
(ii) The pseudo-representation *T* (or rather $\sigma _2$) has Hodge–Tate–Sen weights 0,0,1,1.
(iii) Furthermore, if $\Psi$ is any character that occurs in the decomposition of $T|_{D_p}$ into pseudo-representations then we must have $\Psi |_{I_p} = \epsilon$ or $\Psi |_{I_p}=1$.

#### Proof

For (i) we use the Siegel-ordinarity of the $\sigma _z$ for $z \in Z$ and continuity.

For (ii) we apply [5] Lemma 7.5.12 and deduce that the Hodge–Tate–Sen weights in weight 2 are 0,0,1,1.

For (iii) first note that the statement is clear if $\Psi =\phi _{\beta }$ or $\Psi =\phi _{\beta }^{-1}\epsilon$. So we now consider the case when $\gamma |_{D_p}^{\mathrm{ss}}=\Psi \oplus \Psi '$ for some character $\Psi '$. Part (ii) tells us that $\Psi$ is Hodge–Tate of weight 0 or 1, so equal to a finite order character (not necessarily unramified) or the product of such a character and $\epsilon$. We want to use the crystallinity of $\sigma _z$ for $z\in Z$ to deduce that $\Psi$ is crystalline. Results of Kisin and Bellaïche–Chenevier allow to continue crystalline periods for the smallest Hodge–Tate weight. Note that either $\phi _{\beta }$ or $\phi _{\beta }^{-1}\epsilon$ has the same Hodge–Tate weight as $\Psi$. To be able to attribute the crystalline period to $\Psi$ (rather than $\phi _{\beta }$ or $\phi _{\beta }^{-1}\epsilon$) we use the Siegel-ordinary and *p*-distinguishedness assumptions we made on $\sigma _z$ for $z \in Z$:

As in [6] proof of Theorem 4.3 (which uses geometric Frobenius convention, so considers representations dual to the ones we have here) we consider the sheaf ${\mathcal {M}}$ corresponding to ${\mathcal {O}}(X)[D_p]/\ker {\mathbf {T}}$ (cf. [5] Lemma 4.3.7) defined on an open connected affinoid neighbourhood ${\mathcal {U}}$ of $x_0$. We can quotient ${\mathcal {M}}$ by a subsheaf ${\mathcal {L}}$ corresponding to the maximal submodule on which $D_p$ acts by $\phi _{F_4} \epsilon ^{\kappa _4}$. The quotient sheaf $\widetilde{{\mathcal {M}}/{\mathcal {L}}}$ is generically of rank 3 and its semi-simplification specializes at $x_0$ to $\Psi \oplus \Psi ' \oplus \phi _{\beta }$. As in the proof of [6] Theorem 7.2 Siegel-ordinarity further tells us that $\widetilde{{\mathcal {M}}/{\mathcal {L}}}$ has a torsion-free subsheaf ${\mathcal {N}}$ of generic rank 2 such that the specialisations $\sigma '_z$ at $z \in Z$ are 2-dimensional crystalline representations with Hodge–Tate weights $\kappa _2(z), \kappa _3(z)$ and with crystalline period for the appropriate Hodge–Tate weight, i.e. $D_{\mathrm{cris}}(\sigma '_z)^{\varphi =F_i(z) p^{\kappa _i(z)}} \ne 0$ for $i=2$ or 3. (Note that for $k \in {\mathcal {S}}$ we have $\kappa _2(z_k)=k-2$ and $\kappa _3(z_k)=k-1$.) The semi-simplification of the sheaf ${\mathcal {N}}$ specialized at $x_0$ (which we denote by $\overline{{\mathcal {N}}}^{\mathrm{ss}}_{x_0}:=({\mathcal {N}}_{x_0} \otimes E(x_0))^{\mathrm{ss}}$) equals $\Psi \oplus \Psi '$.

We apply [5] Theorem 3.3.3(i) to the locally free strict transform ${\mathcal {N}}'$ of ${\mathcal {N}}$ along the birational morphism $\pi :X' \rightarrow X$ given by [5] Lemma 3.4.2. This gives $D_{\mathrm{cris}}({\mathcal {N}}'_{x'} \otimes E(x'))^{\varphi =F_i(x')p^{\kappa _i(x')}} \ne 0$ for any $x' \in \pi ^{-1}(x_0)$. By comparing traces one can check (see proof of [5] Lemma 7.8.11) that $({\mathcal {N}}'_{x'} \otimes \overline{{\mathbf {Q}}}_p)^{\mathrm{ss}} \cong ({\mathcal {N}}_{x_0} \otimes \overline{{\mathbf {Q}}}_p)^{\mathrm{ss}}$, and so this implies $D_{\mathrm{cris}}(\overline{{\mathcal {N}}}^{\mathrm{ss}}_{x_0})^{\varphi =F_i(x_0)p^{\kappa _i(x_0)}} \ne 0$.

Since by assumption $F_2(x_0) \ne 0$ (and so also $F_3(x_0)\ne 0$) this means that one of the characters $\Psi$ or $\Psi '$ is crystalline, so equal to a power of the cyclotomic character times a finite order unramified character. As discussed before this power must be 0 or 1. As $T|_{D_p} = T|_{D_p}\circ \tau$ with $\tau (g) = \epsilon (g) g^{-1}$ we get $\Psi \Psi '=\epsilon$. So we are done. $\square$

### Possible splitting types of *T*

Now suppose that *T* is reducible. Then *T* is in one of the following cases:

(i) $T=T_1+T_2 +T_3 +T_4$, where each $T_i$ is a character;
(ii) $T=T_1+ T_2 + T_3$, where $T_1$ and $T_3$ are characters and $T_2$ is an irreducible pseudo-representation of dimension 2 (we refer to this type of splitting as the *Saito–Kurokawa type*);
(iii) $T=T_1+T_2$, where $T_1$, $T_2$ are both irreducible pseudo-representations of dimension 2 (we refer to this type of splitting as the *Yoshida type*);
(iv) $T=T_1 + T_2$, where $T_1$ is an irreducible pseudo-representation of dimension 3 and $T_2$ is a character.

#### Proposition 3.2

Cases (i) and (iv) cannot occur.

#### Proof

Case (i) cannot occur because $\overline{\sigma }_k^{\mathrm{ss}}\cong 1 \oplus \rho \oplus \chi$ for every $k\in {\mathcal {S}}$, so also $\overline{T}= 1 + tr \rho + \chi$ and $\rho$ is irreducible (so also $tr \rho$ is irreducible as a pseudo-representation).

Let us now show that *T* is not as in case (iv). Suppose *T* is as in case (iv). Then $T = \xi + tr \rho _0$, where $\xi : G_{\Sigma } \rightarrow {\mathcal {O}}^{\times }$ is a character and $\rho _0$ is a 3-dimensional irreducible representation. As $T=T\circ \tau$, we must have $\xi |_{I_p} =\epsilon \xi |_{I_p}^{-1}$. This contradicts Lemma 3.1(iii). $\square$

For an ordinary newform $g=\sum _{n=1}^{\infty } a_n(g)q^n$ of weight 2 let *L*(*g*, *s*) denote the standard *L*-function of *g* and let $L_p(g,2)$ be the *p*-adic *L*-value denoted by $L_p^{\mathrm{an}}(g, \omega ^{-1}, T=p)$ in Sect. 2 of [8]. The proof of the following theorem will be given in the next section.

#### Theorem 3.3

Assume $N \ne 1$ and that $\rho |_{G_K}$ is absolutely irreducible for $K={\mathbf {Q}}(\sqrt{(-1)^{(p-1)/2}p})$. Suppose that $L(g,1)L_p(g,2)\ne 0$ for all *p*-ordinary newforms *g* of weight 2 and level dividing *Np* such that $a_{\ell }(g) \equiv tr \rho ({{\,\mathrm{Frob}\,}}_{\ell })$ mod $\varpi$ for all primes $\ell \not \mid Np$. Then *T* is not of Saito–Kurokawa type.

Note that there are only finitely many (possibly none) forms *g* as in Theorem 3.3.

#### Example 3.4

To demonstrate that the conditions in the first sentence of the Theorem can be checked to hold in practice consider $N=5*79$ and $p=3$ and let $\rho$ be the 3-torsion of the elliptic curve with Cremona label 395c1 (see [36, Elliptic Curve 395.a1]). This elliptic curve *E* is semistable, ordinary at 3, and its 3-torsion has an irreducible Galois representation which is ramified at both 5 and 79 (as 3 does not divide the $\ell$-valuations of the minimal discriminant for these two primes). To show that $\rho |_{{\mathbf {Q}}(\sqrt{-3})}$ is absolutely irreducible we can argue as in the proof of [42] Theorem 5.2. Using MAGMA [10] we check that there is only one other weight 2 modular form of level dividing $pN=1185$ congruent modulo primes above 3 to the form corresponding to *E*. This form has level 1185 and corresponds to the elliptic curve with Cremona lavel 1185b1 (see [36, Elliptic Curve 1185.e1]).

By consulting LMFDB [36] we check that both modular forms have non-vanishing central *L*-value. Using the pAdicLseries command in Sage [37] we calculated $L_p(g,2)$ in both cases and checked that the two power series in ${\mathbf {Z}}_3[[T]]$ do not vanish when putting $T=3$.

In Sect. 6 we discuss some conditions that guarantee that *T* is not of Yoshida type either. All these results combined would guarantee that *T* is in fact irreducible, however, the assumptions allowing us to rule out the Yoshida type are quite strong (cf. Remark 6.2).

## Ruling out Saito–Kurokawa type

We keep the notation and assumptions of Sects. 2, 3.1 and Theorem 3.3. In this section we will prove Theorem 3.3. Recall that by assumption (4) we have $\overline{\sigma }_k^{\mathrm{ss}} = 1 \oplus \rho \oplus \chi$ for every $k \in {\mathcal {S}}$. Set $\tau _1=1$, $\tau _2=\rho$, $\tau _3=\chi$. The compactness of $G_{\Sigma }$ guarantees that there exists a $G_{\Sigma }$-stable ${\mathcal {O}}_k$-lattice $\Lambda$ inside the representation space of $\sigma _k$. In other words $\sigma _k$ can be conjugated (over $E_k$) to a representation $\sigma _{k, \Lambda }$ with entries in ${\mathcal {O}}_k$. Its reduction mod $\varpi _k$ has the above semi-simplification. This means that we have a filtration of $G_{\Sigma }$-stable subspaces in the space of $\overline{\sigma }_{k,\Lambda }$ of the form

$$\begin{aligned} 0 \subset V_1 \subset V_2 \subset \overline{\sigma }_{k, \Lambda } \end{aligned}$$

with $V_1\cong \tau _{\gamma (1)}$, $V_2/V_1\cong \tau _{\gamma (2)}$ as well as $\overline{\sigma }_{k,\Lambda }/V_2\cong \tau _{\gamma (3)}$ for some permutation $\gamma \in S_3$. In other words there exists a matrix $\overline{M}=\overline{M}_{\gamma }\in {{\,\mathrm{GL}\,}}_4({\mathbf {F}}_k)$ such that

$$\begin{aligned} \overline{M}\overline{\sigma }_{k, \Lambda }\overline{M}^{-1}\cong \left[ \begin{matrix} \tau _{\gamma (1)} &{} *&{}*\\ &{}\tau _{\gamma (2)} &{} *\\ &{}&{} \tau _{\gamma (3)}\end{matrix} \right] . \end{aligned}$$

Using the fact that the natural map ${\mathcal {O}}_k^{\times }\rightarrow {\mathbf {F}}_k^{\times }$ is surjective we see that ${{\,\mathrm{GL}\,}}_4({\mathcal {O}}_k)\rightarrow {{\,\mathrm{GL}\,}}_4({\mathbf {F}}_k)$ is also surjective, hence we can lift $\overline{M}$ to a matrix $M\in {{\,\mathrm{GL}\,}}_4({\mathcal {O}}_k)$. Then conjugating $\sigma _{k,\Lambda }$ by *M* (or in other words changing an ${\mathcal {O}}_k$-basis of the lattice $\Lambda$, but not changing the lattice itself) we get an (isomorphic over ${\mathcal {O}}_k$) representation $\sigma _{k, \Lambda }$ with the above upper-triangular reduction. So, we can conclude that there exists a lattice $\Lambda$ such that

4.1 $$\begin{aligned} \overline{\sigma }_{k, \Lambda }= \left[ \begin{matrix} \tau _{\gamma (1)} &{} *&{}*\\ &{}\tau _{\gamma (2)} &{} *\\ &{}&{} \tau _{\gamma (3)}\end{matrix} \right] .\end{aligned}$$

Now, for a different lattice $\Lambda '$ we get by the same argument again a representation $\overline{\sigma }_{k, \Lambda '}$ as in (4.1) but possibly with a different $\gamma$. The permutation $\gamma$ need not be uniquely determined by the choice of $\Lambda$ as we do not a priori know that the representation $\overline{\sigma }_{k,\Lambda }$ is non-semi-simple. Nevertheless, given $\Lambda$ such a $\gamma$ always exists (as explained above). So each $\Lambda$ determines a subset $\Gamma (\Lambda )\subset S_3$ of permutations.

### Lemma 4.1

Let $k\in {\mathcal {S}}$. Then there exists a $G_{\Sigma }$-stable lattice $\Lambda$ in the representation space of $\sigma _k$ and $\gamma \in \Gamma (\Lambda )$ with $\gamma (3)=2$ such that

$$\begin{aligned} \overline{\sigma }_{k,\Lambda }= \left[ \begin{matrix} \tau _{\gamma (1)} &{} *_1&{}*_2\\ &{}\tau _{\gamma (2)} &{} *_3\\ &{}&{} \rho \end{matrix} \right] \end{aligned}$$

is indecomposable and $$\left[ \begin{matrix} \tau _{\gamma (2)} &{} *_3\\ &{}\rho \end{matrix} \right] $$ is non-semisimple.

### Proof

Consider the graph ${\mathcal {G}}$ whose vertices are elements of the set ${\mathcal {V}}=\{1, \rho , \chi \}$ and where we draw a *directed* edge from $\rho ' \in {\mathcal {V}}$ to $\rho ''\in {\mathcal {V}}$ if there exists a $G_{\Sigma }$-stable lattice $\Lambda '$ such that $\overline{\sigma }_{k, \Lambda '}$ has a subquotient isomorphic to a non-semi-simple representation of the form $$\left[ \begin{matrix}\rho ' &{} x \\ &{} \rho ''\end{matrix} \right] $$. Then by a theorem of Bellaïche for any two $\rho ', \rho ''\in {\mathcal {V}}$, there exists a directed path from $\rho '$ to $\rho ''$ (see Corollaire 1 in [4]). In particular there must be at least one edge originating at $\rho$ and at least one edge ending at $\rho$. In fact we only use the existence of an edge ending at $\rho$. Hence there exists a lattice $\Lambda$ such that at least one of the following is true:

$$\begin{aligned} \overline{\sigma }_{k,\Lambda } =\left[ \begin{matrix} 1 &{} *_0&{}*\\ &{}\rho &{} *\\ &{}&{} \chi \end{matrix} \right] \quad or \quad \left[ \begin{matrix} \chi &{} *&{}*\\ &{}1 &{} *_0\\ &{}&{} \rho \end{matrix} \right] \quad or \quad \left[ \begin{matrix} \chi &{} *_0&{}*\\ &{}\rho &{} *\\ &{}&{}1\end{matrix} \right] \quad or \quad \left[ \begin{matrix} 1 &{} *&{}*\\ &{}\chi &{} *_0\\ &{}&{} \rho \end{matrix} \right] \end{aligned}$$

with $*_0$ non-trivial (this exhausts all the cases where there is an edge ending at $\rho$).

This proves that either

(i) there exists a lattice $\Lambda$ such that
  $$\begin{aligned} \overline{\sigma }_{k,\Lambda } =\left[ \begin{matrix} \chi &{} a&{}b\\ &{}1 &{} c\\ &{}&{} \rho \end{matrix} \right] \end{aligned}$$
  with $$\left[ \begin{matrix} 1&{} c\\ &{}\rho \end{matrix} \right] $$ non-semi-simple, or
(ii) there exists a lattice $\Lambda$ such that
  $$\begin{aligned} \overline{\sigma }_{k,\Lambda } =\left[ \begin{matrix} 1 &{} a&{}b\\ &{}\chi &{} c\\ &{}&{} \rho \end{matrix} \right] \end{aligned}$$
  with $$\left[ \begin{matrix} \chi &{}c \\ &{}\rho \end{matrix} \right] $$ non-semi-simple, or
(iii) there exists a lattice $\Lambda$ and a permutation $\gamma \in \Gamma (\Lambda )$ with $2=\gamma (2)$ such that
  $$\begin{aligned} \overline{\sigma }_{k,\Lambda } =\left[ \begin{matrix} \tau _{\gamma (1)} &{} a&{}b\\ &{}\tau _{\gamma (2)} &{} c\\ &{}&{} \tau _{\gamma (3)}\end{matrix} \right] \end{aligned}$$
  and $$\left[ \begin{matrix} \tau _{\gamma (1)}&{}a\\ &{} \tau _{\gamma (2)}\end{matrix} \right] $$ is non-semisimple.

First assume that we are in case (i) and suppose that $\overline{\sigma }_{k, \Lambda }$ is decomposable, i.e., that $$\overline{\sigma }_{k, \Lambda }=\left[ \begin{matrix} 1 &{} c \\ &{} \rho \end{matrix} \right] \oplus \chi $$ (recall that the class given by *c* is non-split). As we know that $\overline{\sigma }_{k,\Lambda }$ has a submodule on which $G_{\Sigma }$ operates by $\chi$ we can apply Theorem 4.1 in [8] to obtain a new lattice $\Lambda '$ for which

$$\begin{aligned} \overline{\sigma }_{k,\Lambda '} =\left[ \begin{matrix} \chi &{} *&{}*\\ &{}1 &{} c\\ &{}&{} \rho \end{matrix} \right] \not \cong \left[ \begin{matrix} 1 &{} c \\ &{} \rho \end{matrix} \right] \oplus \chi . \end{aligned}$$

Case (ii) is handled in the same way.

Now suppose that we are in case (iii). Then by Lemma 2.2 there exists a lattice $\Lambda '$ so that with respect to $\Lambda '$ we get

$$\begin{aligned} \overline{\sigma }_{k, \Lambda '}=\left[ \begin{matrix} \tau _{\gamma (3)} &{} * &{}*\\ &{} \tau _{\gamma (1)}&{}a\\ &{}&{} \tau _{\gamma (2)}\end{matrix} \right] . \end{aligned}$$

Defining a new permutation $\gamma '$ by $\gamma '(1)=\gamma (3)$, $\gamma '(2)=\gamma (1)$ and $\gamma '(3)=\gamma (2)$, we thus have a lattice $\Lambda '$ and $\gamma '\in \Gamma (\Lambda ')$ such that

$$\begin{aligned} \overline{\sigma }_{k, \Lambda '}=\left[ \begin{matrix} \tau _{\gamma '(1)} &{} * &{}*\\ &{} \tau _{\gamma '(2)}&{}a\\ &{}&{} \tau _{\gamma '(3)}\end{matrix} \right] \end{aligned}$$

with $$\left[ \begin{matrix} \tau _{\gamma '(2)}&{}a\\ &{} \tau _{\gamma '(3)}\end{matrix} \right] $$ non-semi-simple. If $\overline{\sigma }_{k, \Lambda '}$ is decomposable, then the same argument using Theorem 4.1 in [8] yields yet another lattice (for the same $\gamma '$) for which the representation is indecomposable. Here we have that $2= \gamma '(3)$. $\square$

For $\Lambda$ and $\gamma$ as in Lemma 4.1 we define $\overline{x}_k$ by

$$\begin{aligned} \left[ \begin{matrix} \tau _{\gamma (2)}&{}*\\ &{}\rho \end{matrix} \right] =\left[ \begin{matrix} \tau _{\gamma (2)}&{}\overline{x}_k\\ &{} \rho \end{matrix} \right] . \end{aligned}$$

We note that of course $\overline{x}_k$ depends not only on $\Lambda$ but also on the choice of a basis for $\Lambda$, however, its extension class $[\overline{x}_k]\in H^1({\mathbf {Q}}, {{\,\mathrm{Hom}\,}}(\rho , \tau _{\gamma (2)}))$ does not depend on the choice of basis.

For the rest of the section assume that $T= T_1 + T_2+T_3$ with $T_1, T_2, T_3$ where $\Psi _1:=T_1$ and $\Psi _2:=T_3$ are characters and $T_2$ is two-dimensional and irreducible. We assume that $\overline{\Psi }_1 = 1$, $\overline{\Psi }_2=\chi$ and $\overline{T}_2=tr \rho$. Our goal is to show that these assumptions lead to a contradiction, and thus prove Theorem  3.3. Since $T_2$ is irreducible we get by [35] Theorem 1 that $T_2=tr {\tilde{\rho }}$ for some irreducible 2-dimensional representation ${\tilde{\rho }}: G_{\Sigma } \rightarrow {{\,\mathrm{GL}\,}}_2(E)$ reducing to $\rho$.

### Lemma 4.2

The representation ${\tilde{\rho }}$ is ordinary.

### Proof

By Lemma 3.1 we have $\sigma _2 |_{D_p}^{\mathrm{ss}} = \phi _{\beta }^{-1} \epsilon \oplus \phi _{\beta } \oplus \gamma$, where $\gamma$ is two-dimensional. Since $\beta \not \equiv 1$ mod $\varpi$ by our assumption (5), we cannot have $\Psi _1|_{D_p}, \Psi _2|_{D_p} \in \{ \phi _{\beta }^{-1} \epsilon , \phi _{\beta }\}$. Hence it must be the case that ${\tilde{\rho }}|_{D_p}^{\mathrm{ss}} \cong \phi _{\beta }^{-1}\epsilon \oplus \phi _{\beta }$. Suppose $${\tilde{\rho }}|_{D_p} \cong \left[ \begin{matrix} \phi _{\beta } &{} * \\ 0 &{} \phi _{\beta }^{-1}\epsilon \end{matrix} \right] $$. Note that $\overline{{\tilde{\rho }}}\cong \rho$ is irreducible, so in particular well-defined and we have by assumption (see (2.1)) that $\rho |_{D_p}$ does not have an unramified subrepresentation of dimension 1. Thus neither can ${\tilde{\rho }}|_{D_p}$. Hence we get that $${\tilde{\rho }}|_{D_p} \cong \left[ \begin{matrix} \phi _{\beta }^{-1}\epsilon &{} * \\ 0 &{} \phi _{\beta }\end{matrix} \right] $$ as desired. $\square$

Recall that for every $k\in {\mathcal {S}}$ we write $n_k$ for the largest integer such that $tr \sigma _k \equiv T$ (mod $\varpi ^{n_k}$). Note that under the assumptions from Sect. 3.1 one clearly has $n_k\rightarrow \infty$ as *k* approaches 2 *p*-adically.

### Lemma 4.3

Let $k \in {\mathcal {S}}$, ${\mathcal {J}}=\{\Psi _1, {\tilde{\rho }}, \Psi _2\}$ and let $\Lambda$ be a lattice from Lemma 4.1. Let $\gamma \in \Gamma (\Lambda )$ with $\gamma (3)=2$ and let $\overline{x}_k$ be determined by the pair $(\Lambda , \gamma )$ (and a choice of a basis for $\Lambda$) so that

$$\begin{aligned} \overline{\sigma }_k:= \overline{\sigma }_{k, \Lambda } = \left[ \begin{matrix} \tau _{\gamma (1)} &{} * &{} * \\ &{} \tau _{\gamma (2)} &{} \overline{x}_k \\ &{}&{} \tau _{\gamma (3)}\end{matrix} \right] \end{aligned}$$

is indecomposable with non-semi-simple 3-dimensional quotient $$\left[ \begin{matrix} \tau _{\gamma (2)}&{} \overline{x}_k \\ &{} \tau _{\gamma (3)}\end{matrix} \right] $$ (cf. Lemma 4.1). Then

$$\begin{aligned} \sigma _{k,\Lambda } \cong _{{\mathcal {O}}_k} \left[ \begin{matrix} {\tilde{\tau }}_1 &{} y_k &{} z_k \\ &{} {\tilde{\tau }}_2 &{} x_k \\ &{}&{} {\tilde{\tau }}_3 \end{matrix} \right] \pmod {\varpi ^{n_k}}. \end{aligned}$$

Here ${\tilde{\tau }}_i$ are distinct elements of ${\mathcal {J}}$ and ${\tilde{\tau }}_i = \tau _{\gamma (i)}$ mod $\varpi$ and $x_k = \overline{x}_k$ mod $\varpi _k$. In particular the class $[x_k] \in H^1({\mathbf {Q}}, {{\,\mathrm{Hom}\,}}({\tilde{\tau }}_3, {\tilde{\tau }}_2)\otimes {\mathcal {O}}_k/\varpi ^{n_k})$ has the property that $\varpi ^{n_k-1}[x_k] \ne 0$.

### Proof

This follows from Remarks (a) and (d) in [39] (cf. also Theorem 1.1 in [13]). The last statement follows directly from the fact that the quotient $$\left[ \begin{matrix} \tau _{\gamma (2)} &{} \overline{x}_k \\ &{} \tau _{\gamma (3)}\end{matrix} \right] $$ is not semi-simple. $\square$

### Lemma 4.4

There exists an ordinary newform *g* of weight 2 and level dividing *Np* such that ${\tilde{\rho }} = \rho _g$.

### Proof

We first note that by Serre’s Conjecture (Theorem of Khare-Wintenberger) $\rho$ is modular by a form of weight 2 and level *N*. By Lemma 4.2 we have that $${\tilde{\rho }}|_{D_p} \cong \left[ \begin{matrix} \phi _{\beta }^{-1}\epsilon &{} * \\ 0 &{} \phi _{\beta }\end{matrix} \right] $$, i.e., ${\tilde{\rho }}$ is an ordinary deformation of $\rho$. In particular, its Hodge–Tate weights are 1 and 0. Furthermore, the assumption that $\rho |_{G_K}$ be absolutely irreducible (with *K* as in Theorem 3.3) guarantees that ${\tilde{\rho }}$ is modular by some ordinary newform *g* of weight 2 by a generalization of a theorem of Wiles due to Diamond—see Theorem 5.3 in [17]. The *p*-part of the level of *g* is *p* or 1 (see e.g., Lemma 3.26 in [16]). For primes $\ell \mid N$ the level is at most $\ell$ due to our unipotency assumption (6). Since $\rho$ is ramified at $\ell$ this means that $V_{{{\tilde{\rho }}}}^{I_\ell }$ is 1-dimensional. As we are also assuming that the residual reduction $V_{\rho }^{I_{\ell }}$ is 1-dimensional, the Artin conductors of $\rho$ and ${{\tilde{\rho }}}$ agree (as their valuations are given by $\dim V_{{{\tilde{\rho }}}} -\dim V_{{{\tilde{\rho }}}}^{I_{\ell }}+\mathrm{sw}({{\tilde{\rho }}})$ and $\dim V_{\rho } -\dim V_{\rho }^{I_{\ell }}+\mathrm{sw}(\rho )$, respectively, and $\mathrm{sw}(\rho )=\mathrm{sw}({{\tilde{\rho }}})$ by Serre). The Artin conductor equals $\ell$ since $\rho$ is only tamely ramified at $\ell$ (as we assume $V_{\rho }^{I_{\ell }}$ is 1-dimensional and $\det (\rho )$ is unramified). $\square$

### Remark 4.5

1. The reader may note that if no *g* as in the statement of Theorem 3.3 exists then Lemma 4.4 already gives a contradiction to the assumption that *T* is of Saito–Kurokawa type.
2. Note that if we weakened the unipotency assumption (6) to require it only for primes $\ell \equiv 1 \mod p$ one would obtain modularity by a form of level dividing $N^2p$ in Lemma 4.4. Consequently, Theorem 3.3 would still hold with this weaker unipotency assumption as long as we replace level dividing *Np* by level dividing $N^2p$ in its statement.
3. Similar analyses of reducibility ideals for families approximating holomorphic paramodular Saito–Kurokawa lifts were carried out in [32] and [6] in characteristic zero (necessarily under different assumptions, in particular for $L(g, 1) = 0$). In the following we present arguments working in characteristic *p*. However, it is possible that a characteristic zero approach would also yield our result.

In the following we assume that *E* is large enough to contain the eigenvalues of *g*. Write $V_g$ for the representation space of $\rho _g$ and let $V_g^+\subset V_g$ be the one-dimensional subspace on which $I_p$ acts via $\epsilon$. Let $T_g \subset V_g$ be any $G_{\Sigma }$-stable lattice in $V_g$. The following Lemma follows from the fact that any two $G_{\Sigma }$-stable lattices are homothetic.

### Lemma 4.6

Let $\tau : G_{\Sigma } \rightarrow {{\,\mathrm{GL}\,}}_2(E)$ be residually irreducible. Let $\Lambda , \Lambda '$ be two $G_{\Sigma }$-stable lattices in the representation space of $\tau$. Then $\tau _{\Lambda } \cong \tau _{\Lambda '}$ (over ${\mathcal {O}}$). In other words, $\Lambda$ and $\Lambda '$ are isomorphic as ${\mathcal {O}}[G_{\Sigma }]$-modules.

In particular, the action of $G_{\Sigma }$ on $T_g/\varpi T_g$ (which we denote by $\overline{\rho }_{g, T_g}$) is isomorphic to $\overline{\rho }_g\cong \rho$ as the latter representation is irreducible. Furthermore, by Lemma 4.6 we get that the isomorphism class of the restriction of the action of $G_{\Sigma }$ to $I_p$ on $T_g$ is independent of the choice of $T_g$ inside the representation space of $\rho _g$. More precisely, we have the following result.

### Lemma 4.7

One has $$\rho _{g, T_g}|_{I_p} \cong _{{\mathcal {O}}} \left[ \begin{matrix} \epsilon &{} * \\ &{} 1\end{matrix} \right] .$$

### Proof

By Lemma 4.6 it is enough to show that there exists a $G_{\Sigma }$-stable lattice $\Lambda _0$ such that $$\rho _{g, \Lambda _0}|_{I_p} = \left[ \begin{matrix} \epsilon &{}x \\ &{} 1 \end{matrix} \right] .$$ For this see proof of Proposition 6 of [19]. $\square$

Write $W_g$ for $V_g/T_g \cong \rho _{g,T_g} \otimes E/{\mathcal {O}}$. By Lemma 4.7 we know that there exist rank one free ${\mathcal {O}}$-submodules $T_g^+$ and $T_g^-$ of $T_g$ such that $T_g = T_g^+ \oplus T_g^-$ as ${\mathcal {O}}$-modules and that if $e_1\in T_g^+$ and $e_2\in T_g^-$ form a basis of $T_g$ then in the basis $\{e_1, e_2\}$ one has $$\rho _{g, T_g}|_{I_p} = \left[ \begin{matrix}\epsilon &{} x \\ &{} 1 \end{matrix} \right] $$ with $x \not \equiv 0$ mod $\varpi$ (as $\overline{\rho }_g|_{I_p}=\rho |_{I_p}$ is non-split). One clearly has $T_g^+\otimes _{{\mathcal {O}}}E = V_g^+$. Set $W_g^+:= V_g^+/T_g^+ \cong T_g^+\otimes _{{\mathcal {O}}}E/{\mathcal {O}}$.

Following [32] 3.1.3 we define Greenberg-style Selmer groups

$$\begin{aligned} \mathrm{Sel}_i: = \ker \left( H^1(G_{\Sigma } , W_g\otimes \Psi _i^{-1}) \xrightarrow {\mathrm{res}_{I_p}} H^1(I_p, (W_g/W_g^+) \otimes \Psi _i^{-1})\right) , i=1, 2. \end{aligned}$$

### Lemma 4.8

One has $\Psi _1=1$ and $\Psi _2=\epsilon$.

### Proof

By assumption (6) we know that $\Psi _1$ and $\Psi _2$ are unramified away from *p*. Since $\overline{\Psi }_1=1$ and $\overline{\Psi }_2=\chi$ we know by Lemma 3.1(iii) that $\Psi _1$ is unramified everywhere, hence trivial. As $\Psi _1 \Psi _2=\epsilon$ we get $\Psi _2=\epsilon$. $\square$

### Proposition 4.9

The groups $\mathrm{Sel}_i$, $i=1,2$ are finite.

### Proof

Recall

$$\begin{aligned} L(g, s) =\prod _{\ell \not \mid N} (1-a_{\ell }(g)\ell ^{-s} + \ell ^{-2s+1})^{-1} \prod _{\ell \mid N}(1-a_{\ell }(g)\ell ^{-s})^{-1}\quad for \mathrm{Re}(s)\gg 0. \end{aligned}$$

Let $L^{N}(g,s)$ be defined in the same way but omitting the Euler factors at primes $\ell \mid N$. By Theorem 4.6.17 in [25] we get that the $\ell$-eigenvalue $a_{\ell }(g)$ of *g* equals 0 or $\pm 1$, hence $1-a_{\ell }(g)\ell ^{-i} \ne 0$ for $i=1,2$. This implies that $L(g,i) \ne 0$ if and only if $L^{N}(g, i) \ne 0$ for $i \in \{1,2\}$. By [33] Theorem 3.36 we have $\#\mathrm{Sel}_1 \le \#{\mathcal {O}}/L_{\mathrm{alg}}^N(g,1)$.

In the notation of [33] we are in the case $m=0$ and $a_p(g)-1 \in {\mathcal {O}}^{\times }$ due to our *p*-distinguishedness assumption  2.1 on $\rho$ (which implies that $\rho _{I_p}({{\,\mathrm{Frob}\,}}_p)=\eta ({{\,\mathrm{Frob}\,}}_p) \equiv a_p(g) \not \equiv 1 \mod \varpi$). Note that we assume $N \ne 1$ in Theorem 3.3, so there exists an $\ell$ for which $\rho |_{I_\ell } \ne 1$. As explained in [31] pages 187/8 this (together with $\rho$ irreducible) also makes redundant the assumption in [33] Theorem 3.36 that the image of $\rho _g$ contains $\mathrm{SL}_2({\mathbf {Z}}_p)$.

For $i=2$ we use the argument from the proof of [8] Proposition 2.10: We consider the cyclotomic Main Conjecture of Iwasawa theory for ${{\,\mathrm{GL}\,}}_2$ (in particular the bound proved by [21] Theorem 17.4 with the assumption on the image of $\rho _g$ relaxed as discussed above) for the Teichmueller twist $g \otimes \omega ^{-1}$ and use the control theorem ( [8] Theorem 2.11) to specialize the cyclotomic variable at $T=p$ (corresponding to $s=2$). We deduce that

$$\begin{aligned} \#\mathrm{Sel}_2 \le \# {\mathcal {O}}/L^N_p(g,2). \end{aligned}$$

We note that the assumption in [8] Proposition 2.10 that $p \ne 3$ can be removed as long as $a_p(f)\not \equiv 1$ mod $\varpi$. Let us explain the modifications necessary to the proof of that Proposition (with notation as in [loc.cit.]). We set $g'=g\otimes \omega ^{-1}$ (note that $g'$ is denoted by *g* in [8] and our current *g* is denoted by *f* there) and have

$$\begin{aligned} \rho _{g'}|_{D_p}=\left[ \begin{matrix} \phi \epsilon \omega ^{-1} &{} * \\ &{} \phi ^{-1}\omega ^{-1}\end{matrix} \right] , \end{aligned}$$

where $\phi$ is unramified at *p* with $\phi ({{\,\mathrm{Frob}\,}}_p)=a_p(g)$. This gives us $M[x]^-\cong (E/{\mathcal {O}})(\phi ^{-1}\epsilon ^{-1})$ and $M^-[x]^*(1)=E/{\mathcal {O}}(\phi \epsilon ^{2})$, from which we see that

4.2 $$\begin{aligned} (M^-[x])^*(1)^{I_p}={\left\{ \begin{array}{ll} {\mathbf {F}}(\phi ) &{} p= 3\\ 0&{}p\ne 3\end{array}\right. }\end{aligned}$$

For an arbitrary *p*, we denote by $K=M^-[(x,\varpi )]$ the kernel of multiplication by $\varpi$:

4.3 $$\begin{aligned} 0\rightarrow K \rightarrow M^-[x]\xrightarrow {\cdot \varpi } M^-[x]\rightarrow 0.\end{aligned}$$

From the sequence (4.3) we obtain the corresponding long exact sequence

4.4 $$\begin{aligned}&0\rightarrow K^{D_p} \rightarrow M^-[x]^{D_p}\xrightarrow {\cdot \varpi } M^-[x]^{D_p}\rightarrow H^1({\mathbf {Q}}_p,K)\rightarrow H^1({\mathbf {Q}}_p,M^-[x])\nonumber \\&\quad \xrightarrow {\cdot \varpi } H^1({\mathbf {Q}}_p, M^-[x])\rightarrow H^2({\mathbf {Q}}_p,K). \end{aligned}$$

By [28], Theorem 1.4.1(2) we get

$$\begin{aligned} H^2({\mathbf {Q}}_p,K)\cong {{\,\mathrm{Hom}\,}}(H^0({\mathbf {Q}}_p,K^*(1)),{\mathbf {F}}). \end{aligned}$$

As $K^*(1)={\mathbf {F}}(\phi \omega ^2)$ we see that

4.5 $$\begin{aligned} H^0({\mathbf {Q}}_p,K^*(1))={\left\{ \begin{array}{ll}0&{}if a_p(g)\not \equiv 1 (mod \varpi ) or p\ne 3 \\ {\mathbf {F}}&{}if a_p(g)\equiv 1 (mod \varpi ) and p=3 \end{array}\right. }.\end{aligned}$$

From now on assume that $a_p(g)\not \equiv 1$ (mod $\varpi$) or $p\ne 3$ (note that for the sake of the Proposition we always have $a_p(g)\not \equiv 1$ by our *p*-distinguishedness assumption). Then (4.5) implies that the map $H^1({\mathbf {Q}}_p,M^-[x])\xrightarrow {\cdot \varpi } H^1({\mathbf {Q}}_p, M^-[x])$ is surjective, so $H^1({\mathbf {Q}}_p, M^-[x])$ is $\varpi$-divisible. It follows from the dimension argument in the proof of Lemma 3.18 in [33] that the corank of $H^1({\mathbf {Q}}_p, M^-[x])$ is one hence we conclude that $H^1({\mathbf {Q}}_p, M^-[x])\cong E/{\mathcal {O}}.$

Now consider the inflation-restriction sequence

4.6 $$\begin{aligned}&0 \rightarrow H^1(D_p/I_p, M^-[x]^{I_p})\rightarrow H^1({\mathbf {Q}}_p, M^-[x])\rightarrow H^1(I_p, M^-[x])^{D_p}\nonumber \\&\quad \rightarrow H^2(D_p/I_p, M^-[x]^{I_p}).\end{aligned}$$

The first and the last group are zero since $M^-[x]^{I_p}=(E/{\mathcal {O}})(\epsilon ^{-1})^{I_p}=0$. So, we get

$$\begin{aligned} H^1({\mathbf {Q}}_p, M^-[x])\cong H^1(I_p, M^-[x])^{D_p}. \end{aligned}$$

So, finally we get

$$\begin{aligned} H^1(I_p, M^-[x])^{D_p}=H^1({\mathbf {Q}}_p, M^-[x])=E/{\mathcal {O}}\end{aligned}$$

recovering the conclusion of [33], Lemma 3.18 in this case. With this lemma in place the rest of arguments in Proposition 2.10 of [8] remain unchanged. $\square$

As the representations $\sigma _{k, \Lambda }$ are valued in ${\mathcal {O}}_k$, rather than ${\mathcal {O}}$ we need to introduce some auxiliary Selmer groups. For $k \in {\mathcal {S}}$ and $r \in {\mathbf {Z}}_+$ we set

$$\begin{aligned} \mathrm{Sel}_{i,k,r}: = \ker \left( H^1(G_{\Sigma } , T_{g,k,r}\otimes \Psi _i^{-1}) \xrightarrow {\mathrm{res}_{p}} H^1(I_p, (T_{g,k, r}/T_{g,k, r}^+) \otimes \Psi _i^{-1})\right) , i=1, 2, \end{aligned}$$

where $$T^?_{g, k,r}=T^?_g\otimes {\mathcal {O}}_k/\varpi ^r{\mathcal {O}}_k$$ for $$?\in \{+, \emptyset \}$$.

Note that for $k=2$ (note that ${\mathcal {O}}_2={\mathcal {O}}$) we have a natural map

4.7 $$\begin{aligned} \mathrm{Sel}_{i,2,r} \rightarrow \mathrm{Sel}_i[\varpi ^{r}]\end{aligned}$$

We claim that this map is injective.

We have the following commutative diagram (for $i=1,2$) with exact rows:where *K* is defined as the kernel of the restriction map and recall that $W_g=V_g/T_g$. The map $c\mapsto \varpi ^{-r}c$ gives an isomorphism $T_{g,2,r} \cong W_g[\varpi ^r]$ and then irreducibility of $\overline{\rho }_g$ guarantees that

4.8 $$\begin{aligned} H^1(G_{\Sigma }, W_g\otimes \Psi _i^{-1}[\varpi ^{r}]) = H^1(G_{\Sigma }, W_g\otimes \Psi _i^{-1})[\varpi ^{r}].\end{aligned}$$

This gives the isomorphism on the second vertical arrow. As any $c\in \mathrm{Sel}_{i,2,r}$ viewed inside $H^1(G_{\Sigma }, W_g \otimes \Psi _i^{-1})[\varpi ^{r}]$ via the isomorphism of the middle arrow is killed under the restriction map by commutativity, we conclude that $\mathrm{Sel}_{i,2,r}\subset K$. On the other hand *K* is clearly a subgroup of ${{{\,\mathrm{Sel}\,}}}_i[\varpi ^r]$.

Let $\Lambda$ be a lattice as in Lemma 4.1, let $\gamma \in \Gamma (\Lambda )$ and let $\overline{x}_k$ be determined by $\Lambda$ and $\gamma$ (and a choice of a basis for $\Lambda$). This (after possibly making a change of basis of $\Lambda$ which does not affect the chosen basis of the residual representation) determines $x_k$ as in Lemma 4.3. From now on we fix a basis of $\Lambda$ (which is a certain re-ordering of the basis chosen so far) to ensure a certain convenient order of the diagonal pieces (mod $\varpi ^{n_k}$), namely we want $\Psi _1$ to be first followed by ${\tilde{\rho }}$ and $\Psi _2$. This means that in that basis $\sigma _k$ mod $\varpi ^{n_k}$ may no longer be upper-triangular and in that basis we write

$$\begin{aligned} \sigma _k=\left[ \begin{matrix} \Psi _1 &{} a_k &{} b_k \\ d_k &{} {\tilde{\rho }} &{} c_k \\ e_k &{} f_k &{} \Psi _2 \end{matrix} \right] \pmod {\varpi ^{n_k}} \end{aligned}$$

with $$a_k=\left[ \begin{matrix} a_k^1&a_k^2\end{matrix} \right] $$, $$d_k = \left[ \begin{matrix} d_k^1&d_k^2\end{matrix} \right] ^t$$, $$c_k=\left[ \begin{matrix} c_k^1&c_k^2 \end{matrix} \right] ^t$$ and $$f_k=\left[ \begin{matrix} f_k^1&f_k^2\end{matrix} \right] $$. As $2=\gamma (3)$ (cf. Lemma 4.1), we conclude that $\overline{x}_k=\overline{a}_k$ or $\overline{f}_k$. Indeed, if $\gamma (1)=1$ and $\gamma (2)=3$ then in the basis ${\mathcal {B}}$ of $\Lambda$ that was used to define $\overline{x}_k$ we have

$$\begin{aligned} \overline{\sigma }_{k, {\mathcal {B}}}=\left[ \begin{matrix} 1&{}*&{}*\\ &{} \chi &{}\overline{x}_k\\ &{}&{}\rho \end{matrix} \right] . \end{aligned}$$

By conjugating by an appropriate permutation matrix we obtain

$$\begin{aligned} \overline{\sigma }_{k, {\mathcal {B}}'}=\left[ \begin{matrix} 1&{}*&{}* \\ &{}\rho \\ &{}\overline{x}_k&{}\chi \end{matrix} \right] . \end{aligned}$$

So we get $\overline{x}_k=\overline{f}_k$. If $\gamma (1)=3$ and $\gamma (2)=1$, then in the basis ${\mathcal {B}}$ as above we have

$$\begin{aligned} \overline{\sigma }_{k, {\mathcal {B}}}=\left[ \begin{matrix} \chi &{}*&{}*\\ &{} 1&{}\overline{x}_k\\ &{}&{}\rho \end{matrix} \right] . \end{aligned}$$

So, conjugating by another permutation matrix we obtain

$$\begin{aligned} \overline{\sigma }_{k, {\mathcal {B}}'}=\left[ \begin{matrix} 1&{}\overline{x}_k \\ {} &{}\rho \\ *&{}*&{}\chi \end{matrix} \right] . \end{aligned}$$

In this case we get $\overline{x}_k=\overline{a}_k$.

### Proposition 4.10

If $\overline{x}_k= \overline{f}_k$, then $[x_k] \in \mathrm{Sel}_{1, k, n_k}$. If $\overline{x}_k=\overline{a}_k$, then $[x_k] \in \mathrm{Sel}_{2, k, n_k}$. In either case $\varpi ^{n_k-1}[x_k] \ne 0$.

### Proof

Write

$$\begin{aligned} \sigma _k = \left[ \begin{matrix} \Psi _1 &{} a_k &{} b_k \\ d_k &{} {\tilde{\rho }} &{} c_k \\ e_k &{} f_k &{} \Psi _2 \end{matrix} \right] \pmod {\varpi ^{n_k}} \end{aligned}$$

as before with $$a_k=\left[ \begin{matrix} a_k^1&a_k^2\end{matrix} \right] $$, $$d_k = \left[ \begin{matrix} d_k^1&d_k^2\end{matrix} \right] ^t$$, $$c_k=\left[ \begin{matrix} c_k^1&c_k^2 \end{matrix} \right] ^t$$ and $$f_k=\left[ \begin{matrix} f_k^1&f_k^2\end{matrix} \right] $$. By Siegel-ordinarity we have

$$\begin{aligned} \sigma _k|_{D_p} \cong _{E_k} \left[ \begin{matrix}\phi _{\beta }^{-1} \epsilon &{}*&{}*&{}*\\ &{}*&{}*&{}*\\ &{}*&{}*&{}*\\ &{}&{}&{}\phi _{\beta }\end{matrix} \right] . \end{aligned}$$

Furthermore, by Lemma 4.2 we have $${\tilde{\rho }}|_{D_p} = \left[ \begin{matrix} \phi _{\beta }^{-1} \epsilon &{} h \\ &{} \phi _{\beta } \end{matrix} \right] .$$ Thus in particular

$$\begin{aligned} (\sigma _k|_{D_p} \pmod {\varpi ^{n_k}})^{\mathrm{ss}} = \Psi _1 \oplus \Psi _2 \oplus \phi _{\beta }^{-1} \epsilon \oplus \phi _{\beta } \pmod {\varpi ^{n_k}}. \end{aligned}$$

Conjugating $\sigma _k$ by a permutation matrix we see that

$$\begin{aligned} \sigma _k|_{D_p} \cong _{{\mathcal {O}}_k} \left[ \begin{matrix}\phi _{\beta }^{-1} \epsilon &{} d_k^1 &{} c_k^1 &{} h\\ a_k^1 &{} \Psi _1 &{} b_k &{} a_k^2\\ f_k^1 &{} e_k &{} \Psi _2 &{} f_k^2\\ 0&{}d_k^2&{} c_k^2 &{} \phi _{\beta }\end{matrix} \right] \pmod {\varpi ^{n_k}}. \end{aligned}$$

To complete the proof of Proposition 4.10 we need several lemmas. $\square$

### Lemma 4.11

One has

- If $\overline{x}_k =\overline{a}_k$, then $a_k^1$ gives rise to an extension of $D_p$-modules $$\left[ \begin{matrix} \Psi _1 &{} a_k^1 \\ &{} \phi ^{-1}_\beta \epsilon \end{matrix} \right] $$ mod $\varpi ^{n_k}$, which splits, i.e., $[a_k^1]=0$.
- If $\overline{x}_k =\overline{f}_k$, then $f_k^1$ gives rise to an extension of $D_p$-modules $$\left[ \begin{matrix} \Psi _2 &{} f_k^1 \\ &{} \phi ^{-1}_\beta \epsilon \end{matrix} \right] $$ mod $\varpi ^{n_k}$, which splits, i.e., $[f_k^1]=0$.

### Proof

Assume that $x_k=a_k$, i.e., that $$\sigma _k = \left[ \begin{matrix} \Psi _2 &{} y_k &{} z_k \\ &{} \Psi _1 &{} a_k \\ &{}&{} {\tilde{\rho }}\end{matrix} \right] $$ mod $\varpi ^{n_k}$ as in Lemma 4.3. First note that (after possibly changing to an appropriate basis for the ${\tilde{\rho }}$-piece and using Lemma 4.7) Siegel-ordinarity implies that

4.9 $$\begin{aligned} \sigma _k|_{D_p} = \left[ \begin{matrix} \Psi _2 &{} y_k &{} z_k^1&{}z_k^2 \\ &{} \Psi _1 &{} a_k^1 &{} a_k^2 \\ &{}&{} \phi _{\beta }^{-1}\epsilon &{} h \\ &{}&{}&{} \phi _{\beta } \end{matrix} \right] \quad \pmod {\varpi ^{n_k}}.\end{aligned}$$

Hence we see that there indeed is a rank 2 free ${\mathcal {O}}_k/\varpi ^{n_k}[D_p]$-subquotient $$S=\left[ \begin{matrix} \Psi _1 &{} a_k^1 \\ &{} \phi _{\beta }^{-1}\epsilon \end{matrix} \right] $$ as claimed in the Lemma. It remains to show that *S* splits. Assume it does not. Let *V* be the representation space for $\sigma _k$. By Siegel-ordinarity it has a $D_p$-stable line *L* on which $D_p$ acts via $\phi _{\beta }^{-1}\epsilon$. Let $\Lambda$ be a $G_{\Sigma }$-stable lattice giving $\sigma _k$ such that $\sigma _k|_{D_p}$ mod $\varpi ^{n_k}$ has the form (4.9). Then we see by Lemma 2.1 that this $\Lambda$ must have a $D_p$-stable rank one submodule with $D_p$ action by $\phi _{\beta }^{-1}\epsilon$, hence finally $\Lambda _k:=\Lambda$ mod $\varpi ^{n_k}$ must have a free ${\mathcal {O}}_k/\varpi ^{n_k}$-submodule $\Lambda _0$ of rank one on which $D_p$ acts by $\phi _{\beta }^{-1}\epsilon$.

We now claim that the subquotient *S* also has a free ${\mathcal {O}}_k/\varpi ^{n_k}$-submodule which is stabilized by $D_p$ and on which $D_p$ acts via $\phi _{\beta }^{-1}\epsilon$. Indeed, write ${\mathcal {B}}=\{e_1, \dots , e_4\}$ for an ${\mathcal {O}}_k/\varpi ^{n_k}$-basis of $\Lambda _k$ such that with respect to that basis we have $\sigma _k|_{D_p}$ in form (4.9). Write $\Lambda '=({\mathcal {O}}_k/\varpi ^{n_k})e_1 \oplus ({\mathcal {O}}_k/\varpi ^{n_k}) e_2 \oplus ({\mathcal {O}}_k/\varpi ^{n_k}) e_3$ and $\Lambda '':= ({\mathcal {O}}_k/\varpi ^{n_k})e_4$. We note that $\Lambda '$ is stable under the action of $D_p$. We first want to show that $\Lambda _0 \subset \Lambda '$. Let $v_0 \in \Lambda _0$ be an ${\mathcal {O}}_k/\varpi ^{n_k}$-module generator. Using the fact that ${\mathcal {B}}$ is a basis we can decompose $v_0$ uniquely as $v_0 = v_0' + v_0''$ with $v_0' \in \Lambda '$ and $v_0'' \in \Lambda ''$. We want to show that $v_0''=0$. Let $g \in I_p$ be such that $\chi (g) \ne 1$. Then $g \cdot v_0 = \phi _{\beta }^{-1}\epsilon (g) v_0 = \epsilon (g) v_0$. On the other hand $g\cdot v_0 = g \cdot v'_0 + g\cdot v_0''$. We have that $g \cdot v'_0 \in \Lambda '$ and $g \cdot v_0'' = \phi _{\beta }(g) v_0'' + v' = v_0'' + v'$ for some $v'\in \Lambda '$. So we have

$$\begin{aligned} \epsilon (g) v'_0 + \epsilon (g)v_0''= \epsilon (g) v_0 = g \cdot v_0 = g \cdot v'_0 + v_0'' + v' \implies \epsilon (g)v_0'' - v_0'' \in \Lambda ' \cap \Lambda '' = 0. \end{aligned}$$

Since $\chi (g) \ne 1$, we see that $\epsilon (g)-1 \in ({\mathcal {O}}_k/\varpi ^{n_k})^{\times }$, which implies that $v_0''=0$. So $\Lambda _0 \subset \Lambda '$.

Now set $\Lambda ''=({\mathcal {O}}_k/\varpi ^{n_k})e_1$. This is a $D_p$-stable submodule of $\Lambda '$ on which $D_p$ acts via $\Psi _2$. Notice that we have $S= \Lambda '/\Lambda ''$ as $D_p$-modules. Clearly the image of $\Lambda _0\subset \Lambda '$ in *S* is the desired $D_p$-stable ${\mathcal {O}}_k/\varpi ^{n_k}$-submodule of *S* on which $D_p$ acts via $\phi _{\beta }^{-1}\epsilon$. We just need to show that this image is free of rank one over ${\mathcal {O}}/\varpi ^{n_k}$. Suppose this is not the case, i.e., that $\Lambda _0 \cap \Lambda '' \ne 0$, so $0\ne w_0:=\varpi ^s v_0\in \Lambda ''$ for some $0\le s< n_k$. Let $d \in D_p$ be such that $\Psi _1(d) \not \equiv \phi _{\beta }^{-1}\epsilon (d)$ mod $\varpi$. Then we get $\phi _{\beta }^{-1}\epsilon (d) w_0 = d \cdot w_0 = \Psi _1(d) w_0$, which implies $w_0=0$, a contradiction. This now proves the claim about *S*.

In other words there must exist a matrix $$A=\left[ \begin{matrix} a&{}b \\ c&{} d \end{matrix} \right] \in {{\,\mathrm{GL}\,}}_2({\mathcal {O}}_k)$$ such that

$$\begin{aligned} \left[ \begin{matrix} \Psi _1 &{} a_k^1 \\ &{} \phi _{\beta }^{-1} \epsilon \end{matrix} \right] A = A \left[ \begin{matrix} \phi _{\beta }^{-1} \epsilon &{} * \\ &{} \Psi _1\end{matrix} \right] \pmod {\varpi ^{n_k}}. \end{aligned}$$

Suppose that $[a_k^1]\ne 0$, i.e., that there exists $g \in D_p$ such that $\Psi _1(g)=\phi _{\beta }^{-1}\epsilon (g)=1$ but $a_k^1(g)\ne 0$. Then comparing the upper left entries of both sides evaluated at *g* we get $a+a_k^1(g) c = a$, from which we get that $c\equiv 0$ mod $\varpi$. For the same entry, but for a general element $g'\in D_p$ such that $\phi _{\beta }^{-1} \epsilon (g') \not \equiv \Psi _1(g')$ (mod $\varpi$), we get $\Psi _1(g') a + c a_k^1(g') = a \phi _{\beta }^{-1}\epsilon (g')$. Reducing this equation mod $\varpi$ we thus conclude that $a \equiv 0$ (mod $\varpi$). This is a contradiction since *A* is invertible.

The other case, i.e., where $\overline{x}_k = \overline{f}_k$ is handled similarly using the fact that $\Psi _1|_{D_p}$, $\Psi _2|_{D_p}$, $\phi _{\beta }^{-1}\epsilon$, $\phi _{\beta }$ are all pairwise distinct mod $\varpi$. This finishes the proof of Lemma 4.11. $\square$

We are now ready to complete the proof of Proposition  4.10. Recall that ${\tilde{\rho }} = \rho _g$.

Suppose that $x_k=a_k$ or $x_k=f_k$. In the first case $\sigma _k$ mod $\varpi ^{n_k}$ has a submodule $$\tau = \left[ \begin{matrix} \Psi _1 &{} a_k \\ &{} {\tilde{\rho }}\end{matrix} \right] $$ which is non-split mod $\varpi$ as $[\overline{x}_k]\ne 0$. In the latter case $\sigma _k$ mod $\varpi ^{n_k}$ has a quotient $$\tau = \left[ \begin{matrix} \Psi _2&{} * \\ &{} {\tilde{\rho }} \end{matrix} \right] $$, i.e., $\sigma _k$ mod $\varpi ^{n_k}$ has a quotient $$\tau = \left[ \begin{matrix} {\tilde{\rho }} \\ f_k &{} \Psi _2 \end{matrix} \right] $$ which is non-split mod $\varpi$ as $[\overline{x}_k]\ne 0$. Thus $a_k$ (resp. $f_k$) gives rise to a class in

$$\begin{aligned} H^1(G_{\Sigma }, {{\,\mathrm{Hom}\,}}(T_{g,k,n_k}, {\mathcal {O}}_k/\varpi ^{n_k}{\mathcal {O}}_k(\Psi _i))) \quad for i=1 (resp. i=2) \end{aligned}$$

such that the class is not annihilated by $\varpi ^{n_k-1}$. By Lemma 4.11 we must have $$\tau |_{D_p} = \left[ \begin{matrix} \Psi _1 &{}0&{}a_k^2\\ 0&{} \phi _{\beta }^{-1}\epsilon &{} h \\ 0 &{} 0 &{} \phi _{\beta } \end{matrix} \right] $$ if $x_k=a_k$ and $$\tau |_{D_p}=\left[ \begin{matrix} \phi _{\beta }^{-1}\epsilon &{} h &{} 0\\ 0 &{} \phi _{\beta }&{} 0 \\ 0 &{} f_k^2 &{} \Psi _2\end{matrix} \right] $$ in case $x_k=f_k$.

We now focus on $x_k=a_k$, the other case being analogous. We will show that for every $\gamma \in I_p$ the homomorphism $a_k(\gamma )$ kills $T_{g,k,n_k}^+$. Indeed, in the basis giving rise to $\tau$ as above, the module $T_{g,k,n_k}$ corresponds to vectors $$\left[ \begin{matrix} 0\\ \alpha \\ \beta \end{matrix} \right] $$ while the submodule $T^+_{g,k,n_k}$ of $T_{g,k,n_k}$ corresponds to vectors of the form $$\left[ \begin{matrix} 0\\ \alpha \\ 0 \end{matrix} \right] \in T_{g,k,n_k}$$, as on these vectors $I_p$ acts via $\epsilon$. Note that in the basis which gives the above form of $\tau$ we have $$a_k = \left[ \begin{matrix} 0&a_k^2\end{matrix} \right] $$, while $T_{g,k,n_k}^+$ is given again by the vectors of the form $$\left[ \begin{matrix} 0\\ \alpha \\ 0 \end{matrix} \right] \in T_{g,k,n_k}$$.

By the discussion above we conclude that the inverse of the isomorphism $\psi : {\mathcal {O}}_k/\varpi ^{n_k}(\Psi _1) \otimes T_{g,k,n_k}^{\vee } \rightarrow {{\,\mathrm{Hom}\,}}(T_{g,k,n_k}, {\mathcal {O}}_k/\varpi ^{n_k}(\Psi _1))$ carries $a_k(\gamma )$ into the subspace ${\mathcal {O}}_k/\varpi ^{n_k}(\Psi _1) \otimes (T_{g,k,n_k}^+)'\subset {\mathcal {O}}_k/\varpi ^{n_k}(\Psi _1)\otimes T_{g,k,n_k}^{\vee }$, where as above $(T_{g,k,n_k}^+)'$ denotes the submodule of $T_{g,k,n_k}^{\vee }$ consisting of functionals which kill $T_{g,k,n_k}^+$.

Note that since $\Psi _1\Psi _2=\epsilon$, we get $\Psi _1\otimes \rho _g^{\vee } \cong \Psi _2^{-1}\epsilon \otimes \rho _g^{\vee } \cong \Psi _2^{-1} \otimes \rho _g^{\vee }(1)$. Under these isomorphisms the module ${\mathcal {O}}_k/\varpi ^{n_k}(\Psi _1) \otimes (T_{g,k,n_k}^+)'$ gets mapped to ${\mathcal {O}}_k/\varpi ^{n_k}(\Psi _2^{-1} \epsilon )\otimes (T_{g,k,n_k}^+)'$ and finally to ${\mathcal {O}}_k/\varpi ^{n_k}(\Psi _2^{-1}) \otimes (T_{g,k,n_k}^+)'(1)$. Finally (by essential self-duality of $\rho _g$) there is an isomorphism of $G_{\Sigma }$-modules $\psi ':\rho _g\rightarrow \rho _g^{\vee }(1)$. We note that $T_{g,k,n_k}^+$ is the unique direct summand of $T_{g,k,n_k}$ which is stable under $I_p$ and such that $I_p$ acts on it by $\epsilon$. Hence $\psi '$ (as it is $G_{\Sigma }$-equivariant) must carry $T_{g,k,n_k}^+$ onto the unique direct summand of $T_{g,k,n_k}^{\vee }(1)$ with the same property, i.e., $\psi '(T_{g,k,n_k}^+) = X \otimes \epsilon$ where *X* is the unique direct summand of $T_{g,k,n_k}^{\vee }$ on which $I_p$ acts trivially.

Let $\phi \in (T_{g,k,n_k}^+)'$. Let $\gamma \in I_p$, $$v=\left[ \begin{matrix} v_1 \\ v_2 \end{matrix} \right] \in T_{g,k,n_k}$$. (We suppress the 0 from $$\left[ \begin{matrix} 0\\ v_1 \\ v_2 \end{matrix} \right] $$.) Then

$$\begin{aligned} (\gamma \cdot \phi )(v) = \phi (\rho _g(\gamma ^{-1})v)= \phi \left( \left[ \begin{matrix} \epsilon (\gamma )^{-1} &{} h(\gamma ^{-1})\\ &{} 1\end{matrix} \right] v\right) =\phi \left( \left[ \begin{matrix} \epsilon (\gamma )^{-1} v_1 + h(\gamma ^{-1}) v_2 \\ v_2 \end{matrix} \right] \right) \\ =\phi \left( \left[ \begin{matrix} \epsilon (\gamma )^{-1} v_1 + h(\gamma ^{-1}) v_2-v_1 \\ 0 \end{matrix} \right] +v\right) = \phi (v).\end{aligned}$$

Hence $I_p$ acts trivially on $(T_{g,k,n_k}^+)'$, i.e., we must have $X=(T_{g,k,n_k}^+)'$. In other words $\psi '$ carries $T_{g,k,n_k}^+$ onto $(T_{g,k,n_k}^+)'(1)$. This proves that for $\gamma \in I_p$ we have that $a_k(\gamma )$ is mapped under $\psi ^{-1}$ into ${\mathcal {O}}_k/\varpi ^{n_k}(\Psi _1)\otimes (T_{g,k,n_k}^+)' \cong {\mathcal {O}}_k/\varpi ^{n_k}(\Psi _2^{-1}) \otimes (T_{g,k,n_k}^+)'(1)$ and further mapped under $(\psi ')^{-1}$ into the the direct summand ${\mathcal {O}}_k/\varpi ^{n_k}(\Psi _2^{-1})\otimes T_{g,k,n_k}^+\subset {\mathcal {O}}_k/\varpi ^{n_k}(\Psi _2^{-1})\otimes T_{g,k,n_k}$. Hence we get $[a_k] \in {{\,\mathrm{Sel}\,}}_{2, k, n_k}$.

The case $\overline{x}_k = \overline{f}_k$ is handled in an analogous way. Finally the fact that $\varpi ^{n_k-1}[x_k]\ne 0$ follows from Lemma 4.3. $\square$

### Corollary 4.12

If $\overline{x}_k= \overline{f}_k$, then there exists an element $x'_k \in \mathrm{Sel}_1$ such that $\varpi ^{n_k-1}x'_k \ne 0$. If, on the other hand, $\overline{x}_k=\overline{a}_k$, then there exists an element $x'_k \in \mathrm{Sel}_2$ such that $\varpi ^{n_k-1}x'_k \ne 0$.

### Proof

First note that as the formation of Selmer groups commutes with direct sums of Galois modules and ${\mathcal {O}}_k/\varpi ^r =({\mathcal {O}}/\varpi ^r)^{s}$ where $s= [{\mathcal {O}}_k:{\mathcal {O}}]$ one has $\mathrm{Sel}_{i, k, n_k} = \left( \mathrm{Sel}_{i, 2, n_k}\right) ^s$. If $\overline{x}_k= \overline{f}_k$ then by Proposition 4.10 we get that $[x_k] \in \mathrm{Sel}_{1, k, n_k}$ is such that $\varpi ^{n_k-1} [x_k] \ne 0$. Thus there must exist an element $x'_k \in \mathrm{Sel}_{1, 2, n_k}$ which is not annihilated by $\varpi ^{n_k-1}$. As we have an inclusion $\mathrm{Sel}_{1, 2, n_k} \hookrightarrow \mathrm{Sel}_1[\varpi ^{n_k}]$, we can regard $x_k'$ as an element of $\mathrm{Sel}_1$ which is not killed by $\varpi ^{n_k-1}$. The other case is analogous. $\square$

We are now ready to finish the proof of Theorem 3.3, i.e., that the pseudo-representation *T* is not of Saito–Kurokawa type. Indeed, we will now arrive at a contradiction. Since by Lemma 4.1 for every $k \in {\mathcal {S}}$ there exists $\overline{x}_k \in \{\overline{a}_k, \overline{f}_k\}$ such that $[\overline{x}_k]$ gives rise to a non-split extension of the corresponding Jordan–Holder blocks of $1 \oplus \rho \oplus \chi$, there exists $A\in \{a,f\}$ and an infinite subsequence ${\mathcal {T}}\subset {\mathcal {S}}$ such that for all $k \in {\mathcal {T}}$ we have that $[\overline{x}_k]=[\overline{A}_k]$ is such a non-split extension. Fix such an *A*. Then Proposition 4.10 gives us an extension $[A_k] \in \mathrm{Sel}_{i, k, n_k}$ for $i=1$ or 2 such that $\varpi ^{n_k-1} [A_k] \ne 0$. Set $i(A)=1$ if the extension $[A_k]$ lies in ${{\,\mathrm{Sel}\,}}_{1, k, n_k}$ and $i(A)=2$ if the extension $[A_k]$ lies in ${{\,\mathrm{Sel}\,}}_{2,k, n_k}$. Then by Corollary 4.12 we get an element $A'_k\in \mathrm{Sel}_{i(A)}$ not annihilated by $\varpi ^{n_k-1}$. As $n_k$ tends to $\infty$ for $k \in {\mathcal {T}}$, we see that ${{\,\mathrm{Sel}\,}}_{i(A)}$ must be infinite. Thus we obtain a contradiction to Proposition 4.9.

## Siegel modular forms and paramodular conjecture

In this section, which is an interlude and not part of the logical sequence of the paper, we discuss some automorphic results and a potential application to the Paramodular Conjecture to motivate the results of this paper.

### Siegel modular forms

We recall some facts about Siegel modular forms and their associated Galois representations. By Arthur’s classification (see [3] and [18]) cuspidal automorphic representations for $\mathrm{GSp}_4({\mathbf {A}}_{{\mathbf {Q}}})$ fall into different types. Cuspidal automorphic representations whose transfer to ${{\,\mathrm{GL}\,}}_4$ stays cuspidal are called of “general type” or type (G).

One can attach *p*-adic Galois representations to algebraic automorphic representations $\pi$ for certain $\pi _\infty$ (e.g. holomorphic limit of discrete series). For type (G) representations these Galois representations are expected to be irreducible (see [41] for a summary of what’s known and results in the low weight case). Other types in the classification are known to be associated to reducible *p*-adic Galois representations, see [11] Lemma 2.9.1. Particular examples of such types are the Saito–Kurokawa lifts and Yoshida lifts of elliptic modular forms, whose associated Galois representations have trace of Saito–Kurokawa or Yoshida type respectively. Schmidt [30] proved that holomorphic Siegel modular forms of paramodular level are either of type (G) or Saito–Kurokawa lifts, while other CAP types or Yoshida lifts do not occur.

We denote by $U_{p,1}$ (resp. $U_{p,2}$) the Hecke operators associated to $\mathrm{diag}(1,1,p,p)$ (resp. $\mathrm{diag}(1,p,p^2,p)$). For $\pi$ of sufficiently high weight (i.e. corresponding to classical Siegel eigenforms of weights $k_1 \ge k_2 \ge 3$) we have the following result about properties of the associated Galois representations (for a more detailed statement see [11] Theorem 2.7.1):

#### Theorem 5.1

(Laumon, Weissauer, Sorensen, Mok, Faltings-Chai, Urban) Suppose $\pi$ is a cuspidal automorphic representation for ${{\,\mathrm{GSp}\,}}_4({\mathbf {A}}_{{\mathbf {Q}}})$ of weight $k_1 \ge k_2 \ge 3$. Then there is a continuous semi-simple representation $\rho _{\pi }: G_{{\mathbf {Q}}} \rightarrow {{\,\mathrm{GSp}\,}}_4(\overline{{\mathbf {Q}}}_p)$ with

$$\begin{aligned} \rho _{\pi }^{\vee } \cong \rho _{\pi }(3-k_1-k_2) \end{aligned}$$

satisfying the following properties:

1. For each prime $\ell \ne p$ we have local-global compatibility up to semi-simplification with the local Langlands correspondence proved by Gan-Takeda. In particular, if $\pi$ is unramified at $\ell$ then so is $\rho _{\pi }$ and if $\pi$ is of Iwahori level at $\ell$ then $\rho _{\pi }|_{I_\ell }$ is unipotent.
2. If $\rho _{\pi }$ is irreducible then for each prime $\ell \ne p$ one has local-global compatibility up to Frobenius semi-simplification.
3. $\rho _{\pi }|_{D_p}$ is de Rham with Hodge–Tate weights $k_1+k_2-3, k_1-1, k_2-2, 0$.
4. Assume that $\pi$ is Siegel-ordinary at *p* (i.e $\lambda _{p,1}$ is a *p*-adic unit, $\lambda _{p,2}$ has finite *p*-valuation, where $\lambda _{p,i}$ is the $U_{p,i}$-eigenvalue of $\pi$ for $i=1,2$), then $\rho _{\pi }|_{D_p}$ is Siegel-ordinary in the sense of Definition 2.4 with the unramified character having $\lambda _{p,1}$ as value at ${{\,\mathrm{Frob}\,}}_p$.
5. If $\pi$ is unramified at *p* then the *p*-adic representation $\rho _{\pi }$ is crystalline at *p*. If $\pi$ is also Siegel-ordinary then the characteristic polynomial of Frobenius acting on $D_{\mathrm{cris}}(\rho _{\pi }|_{D_p})$ equals the Hecke polynomial. In particular, the eigenvalues are
  $$\begin{aligned} \lambda _{p,1}, \lambda _{p,1}^{-1}\lambda _{p,2} p^{k_2-2}, \lambda _{p,1}\lambda _{p,2}^{-1}p^{k_1-1}, \lambda _{p,1}^{-1}p^{k_1+k_2-3}. \end{aligned}$$

Suppose now that $\rho$ as in Sect. 2 equals $\overline{\rho }_f$ for $f \in S_2(Np)$. If *f* is ordinary it lies in a Hida family of eigenforms $f_k$. Brown et al. [1, 12, 14] then prove that there exist holomorphic Siegel modular eigenforms $F_k$ for $k \in {\mathcal {S}}$ with ${\mathcal {S}}$ as in Sect. 3 of Iwahori level *N* (level $\Gamma _0^{(2)}(N)$ or $\Gamma _{\mathrm{para}}(N)$ ) that are congruent to the Saito–Kurokawa lifts $SK(f_k)$ modulo $\varpi$ and $\sigma _{F_k}$ is irreducible (see e.g. [1] Corollary 7.5). We expect to be able to prove that we can take these eigenforms to be Siegel ordinary and then the theorem above shows that the associated Galois representations $\sigma _{F_k}$ satisfy the conditions (1)–(6) in Sect. 3.1. To establish that the $tr \sigma _{F_k}$ interpolate *p*-adically is work in progress.

The pseudo-representation of the (Siegel-ordinary, tame level *N*) eigenvariety (see [32] and [2]) would then give rise to ${\mathbf {T}}:G_{\Sigma } \rightarrow {\mathcal {O}}(X)$ for an affinoid *X* containing the limit point $x_0$ of weight (2, 2). One obtains a Zariski dense subset $Z\subset X$ of classical points that are old at *p* such that $(X, {\mathbf {T}}, \{\kappa _n\}, \{F_n\}, Z)$ is a refined family in the sense of Bellaïche–Chenevier. By the above theorem the function $F_1=F_4^{-1}$ interpolates the $U_{p,1}$-eigenvalue $\lambda _{p,1}$, $F_2=F_3^{-1}$ interpolates $\lambda _{p,1}^{-1}\lambda _{p,2}$, so our assumption $F_2(x_0)\ne 0$ would correspond to the $U_{p,2}$-slope of the limit form being finite.

### Discussion of applicability to the paramodular conjecture

For an elliptic modular form *f* of weight $2k-2$ a holomorphic Saito–Kurokawa lift exists under the following conditions on *f* and *k*: for $\Gamma _0^{2}(N)$-level *k* has to be even, for $\Gamma _{\mathrm{para}}(N)$-level the sign of the functional equation of *f* has to be $-1$ (see [29]).

Suppose $\rho =\overline{\rho }_f$ for an ordinary newform *f* of level *N*. For Theorem 3.3 we need to assume that $L(f,1) \ne 0$. Continuing our discussion from the introduction about Saito–Kurokawa congruences, we note that in the case that $L(f,1) \ne 0$ we would therefore need to consider congruences with holomorphic $\Gamma _0^{2}(N)$-level Saito–Kurokawa lifts. However, a different method to the one used by Brown et al. (pointed out to us by Pol van Hoften) could be used to prove the required congruences for paramodular level: Using the arguments from the proof of [34] Theorem D one should be able to prove congruences for the generic (as opposed to the holomorphic) Saito–Kurokawa lift, for which the conditions on *k* and the root number are reversed.

Once the congruence between the generic Saito–Kurokawa lift and a type (G) form has been proved, one could then switch to the holomorphic element of the same packet. If such a congruence could be proved in weight 2 this would also explain the example of the abelian surface of conductor 997 mentioned in [8] (which involves an elliptic modular form *f* with root number $\epsilon =1$ and $L(f,1)=0$).

To demonstrate that examples with $L(f,1) \ne 0$ occur when studying the modularity of abelian surfaces we thank Andrew Sutherland for providing us with the following abelian surface: Let *A* be the Jacobian of the genus 2 curve

$$\begin{aligned} C: y^2 + (x + 1)y = -2x^6 + x^5 - x^4 + 9x^3 - 2x^2 + 2x - 9 \end{aligned}$$

(see [36, Genus 2 Curve 1870.a] and [9]). Then *A* has conductor $1870 = 2*5*11*17$ and comparing values on ${{\,\mathrm{Frob}\,}}_{\ell }$ for $\ell <10^6$ strongly suggests that

$$\begin{aligned} A(\overline{{\mathbf {Q}}})[3] \cong 1 \oplus \overline{\rho }_f \oplus \chi \end{aligned}$$

for *f* the unique weight 2 newform of level $\Gamma _0(17)$ corresponding to the isogeny class of rank 0 elliptic curves over ${\mathbf {Q}}$ with conductor 17.

## Ruling out Yoshida type

Recall that $\sigma _2$ is the representation associated with *T* (cf. Sect. 3.1). In this section we work under the assumptions of Sect. 3 and show that $\sigma _2$ is not the direct sum of two irreducible two-dimensional representations under some additional assumptions.

For a positive integer *N* we will write $S_2^{(2)}(\Gamma ^{\mathrm{para}}(N))$ for weight 2 genus 2 Siegel modular forms of paramodular level *N*.

### Proposition 6.1

Suppose at least one of the following holds:

(I) One has $\ell \not \equiv \pm 1$ mod *p* for all $\ell \mid N$ and $\sigma _2$ is Borel-ordinary at *p*,
(II) One has $\ell \not \equiv \pm 1$ mod *p* for all $\ell \mid N$ and $\sigma _2$ is crystalline at *p*.
(III) One has $p>3$ and $\sigma _2=\sigma _F$ for some classical Siegel modular form $F \in S_2^{(2)}(\Gamma ^{\mathrm{para}}(N))$ which has distinct roots for its Hecke polynomial at *p*.

Then $\sigma _2$ is not of Yoshida type.

### Proof

Assume that in fact $\sigma _2 = \rho _1 \oplus \rho _2$ with $\rho _1, \rho _2$ irreducible and $\overline{\rho }_1=\rho$ and $\overline{\rho }_2^{\mathrm{ss}} = 1 \oplus \chi$. By Lemma 3.1(i) we have $(\sigma _2|_{D_p})^{\mathrm{ss}}=\phi _{\beta }^{-1}\epsilon \oplus \phi _{\beta } \oplus \gamma$ , which as in Lemma 4.2 implies that $\rho _1$ is ordinary, i.e., that $$\rho _1|_{D_p} \cong _E \left[ \begin{matrix} \phi _{\beta }^{-1} \epsilon &{} * \\ &{} \phi _{\beta }\end{matrix} \right] $$. By Lemma 3.1(ii) the Hodge–Tate–Sen weights of $\sigma _2$ are 0,0,1,1.

Proof of (I): As $\sigma _2$ is Borel-ordinary, this forces $\rho _2|_{D_p}$ to be ordinary, i.e., $$\rho _2|_{D_p} \cong \left[ \begin{matrix} \phi _{\alpha }^{-1}\epsilon &{}* \\ &{} \phi _{\alpha }\end{matrix} \right] $$ for some $\alpha \in {\mathcal {O}}^{\times }$. On the other hand since $\rho _2$ is irreducible there exists a $G_{\Sigma }$-stable lattice $\Lambda$ in the space of $\rho _2$ such that with respect to that lattice we have

6.1 $$\begin{aligned} \overline{\rho }_{2, \Lambda } =\left[ \begin{array}{ll} 1 &{} a \\ &{} \chi \end{array} \right] \not \cong 1 \oplus \chi .\end{aligned}$$

By Lemma 2.1, the lattice $\Lambda$ must have a $D_p$-stable line on which $D_p$ acts via $\phi _{\alpha }^{-1}\epsilon$, so $$\overline{\rho }_{2, \Lambda }|_{D_p} \cong \left[ \begin{array}{ll} \overline{\phi }_{\alpha }^{-1} \chi &{} * \\ &{} \overline{\phi }_{\alpha } \end{array} \right] $$. By comparing with the form (6.1) and using that $\chi$ is ramified we conclude that $\overline{\phi }_{\alpha }=1$, so in fact $$\overline{\rho }_{2, \Lambda }|_{D_p} \cong \left[ \begin{array}{ll} \chi &{} * \\ &{} 1 \end{array} \right] $$. Thus $\overline{\rho }_2|_{D_p} \cong 1 \oplus \chi$. This in particular implies that $\overline{\rho }_2$ splits when restricted to $I_p$. Hence *a* gives rise to a class in

$$\begin{aligned} H^1_{\Sigma }({\mathbf {Q}}, {\mathbf {F}}(-1)):=\ker (H^1(G_{\Sigma }, {\mathbf {F}}(-1)) \overset{\mathrm{res}_{p}}{\rightarrow } H^1(I_p, {\mathbf {F}}(-1))). \end{aligned}$$

Since $\ell \not \equiv \pm 1$ mod *p* for all $\ell \mid N$ we use Lemma 6.3 in [7] to conclude that $H^1_{\Sigma }({\mathbf {Q}}, {\mathbf {F}}(-1)) =\ker (H^1(G_{\Sigma }, {\mathbf {F}}(-1)) \rightarrow \prod _{\ell \in \Sigma } H^1(I_{\ell }, {\mathbf {F}}(-1)))$. This part of the class group of ${\mathbf {Q}}(\mu _p)$ is zero by Proposition 6.16 in [40]. This implies that $\overline{\rho }_{2, \Lambda }$ is split which leads to a contradiction.

Proof of (II): As before there exists a $G_{\Sigma }$-stable lattice $\Lambda$ such that with respect to that lattice we have $$\overline{\rho }_{2, \Lambda } =\left[ \begin{array}{ll} 1 &{} a \\ &{} \chi \end{array} \right] \not \cong 1 \oplus \chi $$. Since $\sigma _2$ is crystalline and its Hodge–Tate–Sen weights are 0,0,1,1, it is in the Fontaine–Laffaille range. Hence so is $\rho _2$. This implies (see e.g. [7] Lemma 6.1) that the extension given by *a* gives rise to a non-zero element in $H^1_{\Sigma }({\mathbf {Q}}, {\mathbf {F}}(-1))$, which again gives a contradiction as $H^1_{\Sigma }({\mathbf {Q}}, {\mathbf {F}}(-1))=0$.

Proof of (III): We have $\sigma _2=\sigma _F$ for some classical Siegel modular form $F \in S_2^{(2)}(\Gamma ^{\mathrm{para}}(N))$. We can assume that *F* is not a Saito–Kurokawa lift (as then $tr \sigma _F$ would not be of Yoshida type). By [30] this means that *F* is of type (*G*). The assumption on the roots of the Hecke polynomial implies by [20] Theorem 4.1 or [26] Proposition 4.16 that $\sigma _2$ is crystalline at *p*. If $\ell \not \equiv \pm 1$ mod *p* for all $\ell \mid N$ then we get a contradiction as in (I) and (II). Without this assumption we argue as in the proof of [8] Theorem 8.6, i.e. apply [27] Theorem C and [23] Theorem 7.1 to deduce that *F* would have to be of Yoshida type, i.e. not of type (G), a contradiction. $\square$

### Remark 6.2

Note that the key issue in the Yoshida case is ruling out that $\sigma _2$ is the sum of an (ordinary) 2-dimensional Galois representation associated to a classical form (with associated $\mod p$-representation $\rho$) and a 2-dimensional Galois representation that is a priori not de Rham.

It is worth noting that whilst we are able to rule out that $\sigma _2$ is of Saito–Kurokawa type only using properties of the representations $\sigma _k$ for $k \in {\mathcal {S}}$ the Yoshida type case requires additional information about $\sigma _2$. In particular, while for both the Saito–Kurokawa and the Yoshida type we assume crystallinity of the representations $\sigma _k$, in case (II) of Proposition 6.1 we also need to assume that $\sigma _2$ itself is crystalline. On the other hand, work in progress by Ariel Weiss shows that a classical Siegel-ordinary type (G) eigenform has irreducible Galois representation. This would allow us to drop the assumption in (III) on the distinctness of the roots of the Hecke polynomial.
